# Evolution of repressive sequences within an enhancer contributed to morphological diversity in crucifer plants

**DOI:** 10.1073/pnas.2515732122

**Published:** 2025-12-16

**Authors:** Alessandro Popoli, Remco A. Mentink, Lisa Brombach, Nora Papadima-Karanikou, Manuel Buendia-Monreal, Mingming Fang, Manas Joshi, Saiko Yoshida, Stefan Laurent, Peter Huijser, Miltos Tsiantis

**Affiliations:** ^a^Department of Comparative Development and Genetics, Max Planck Institute for Plant Breeding Research, Cologne 50829, Germany

**Keywords:** leaf shape, evo devo, enhancer, homeobox gene, gene duplication

## Abstract

*Cis*-regulatory sequences, such as enhancers, play a crucial role in morphological evolution, but how their diversification leads to evolution of novel expression patterns that underpin diversity is still poorly understood. Recent work suggests that the function of enhancers is influenced by their genomic sequence context more than previously thought, further complicating our understanding of their contribution to diversity. To address these issues, we investigated the regulation of the *RCO* homeobox gene, which contributed to the evolution of complex leaves following its tandem duplication from *LMI1*. By characterizing seventeen regulatory alleles at the *LMI1*/*RCO* locus that we generated by genome editing, we found that the evolved *RCO* enhancer is subject to pronounced negative regulation that helps delimit the *RCO* expression domain. While the *LMI1* enhancer was strictly required for *LMI1* gene expression, the same was not true for the *RCO* enhancer, which caused only partial loss of function when deleted. We mapped both positively and negatively acting sequences within the *RCO* enhancer, validated them in reporter gene assays and showed that a repressive sequence arose in association with a nested duplication within the evolved *RCO* enhancer. This repressive sequence played a key role in shaping the specific *RCO* expression domain that underlies its role in leaf complexity, and we provide evidence that it helped to prevent potential pleiotropic effects arising from evolutionary diversification or *RCO* expression. Our findings highlight the regulatory features of a diversity-linked enhancer and show that the evolution of repressive sequences is a powerful force in regulatory evolution.

## Significance

Enhancers are regulatory DNA sequences that control gene expression and drive morphological evolution. Yet, how they diversify to create new expression domains remains unclear. We explore this by investigating an enhancer of the *RCO* plant homeobox gene, which arose through gene duplication and promotes leaf complexity. Compared to its ancestral counterpart, the *RCO* enhancer is less stringently required for *RCO* expression and is subject to more repression. This repression helped establish *RCO’s* expression pattern while avoiding potentially pleiotropic effects arising from its change in expression. Our work highlights regulatory features of a diversity-linked enhancer and underscores the importance of repressive sequences in morphological evolution.

A key problem in biology is to identify the genetic basis for morphological differences between species. Diversification of *cis*-regulatory sequences such as enhancers has emerged as a major mechanism involved in morphological evolution (1–5). However, specific examples of how enhancers evolve to yield novel gene expression domains that underpin morphological diversity are lacking. The textbook paradigm of enhancers (6, 7) considers them to be discrete and highly modular, but recent work suggests that they can be strongly entangled with neighboring regulatory sequences (8) further complicating analyses of how enhancers diversify. CRISPR/Cas9 mutagenesis has opened up new opportunities to address these questions (9). However, although genomic areas that contribute to developmental gene regulation have been defined (10–13), CRISPR/Cas9 has not yet been systematically deployed to identify specific mechanisms through which enhancers functionally diversify to produce novel gene expression domains and morphological variation.

**Fig. 1.**
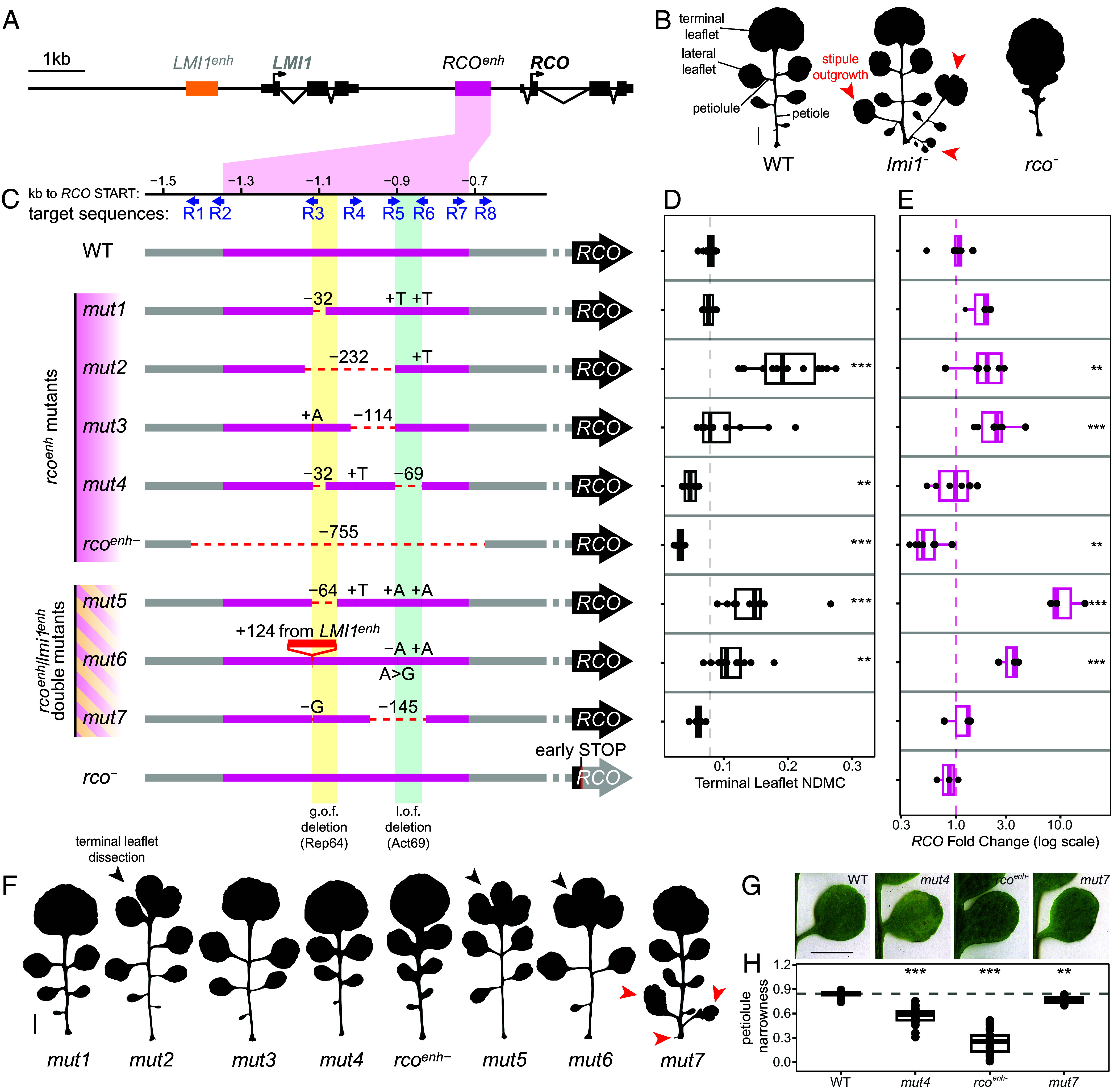
Mutations in the *RCO* enhancer result in weak *RCO* loss-of-function or *RCO* gain-of-function phenotypes. (*A*) Schematic representation of the *LMI1*/*RCO* locus in *C. hirsuta*. (*B*) Silhouettes of representative leaf 5 from wild-type (WT), *lmi1^−^*, and *rco^−^* reference alleles. The leaf-like organs resulting from the stipule outgrowths in *lmi1^−^* are indicated with red arrowheads. (Scale bar: 1 cm.) (*C*) Schematic representation of the CRISPR-Cas9-generated mutant alleles in the *RCO* enhancer. Blue arrows indicate the position and direction of the sgRNA target sequences used. The first five alleles indicate *rco^enh^* mutants and the remaining three indicate *rco^enh^*/*lmi1^enh^* double mutants. Yellow- and green-shaded boxes highlight sequence the deletion of which causes *RCO* gain-of-function (yellow, Repressor 64 or Rep64) or loss-of-function (green, Activator 69 or Act69), respectively. The function of these sequences is also tested in transgenic reporter gene assays in Fig. 4 *D* and *E*. (*D*) Normalized-Differential Margin Complexity (NDMC) of the terminal leaflet (TL) of leaf 5 from the mutants shown in (*C*). Increasing values indicate increasing complexity. The dashed line represents the WT average value. WT: n = 25, *mut1*: n = 13, *mut2*: n = 14, *mut3*: n = 13, *mut4*: n = 11, *rco^enh−^*: n = 18, *mut5*: n = 13, *mut6*: n = 12, *mut7*: n = 7. (*E*) qPCR assay of the relative *RCO* expression levels of the alleles shown in (*C*), measured in 14-d-old seedling apices. The dashed line indicates average expression in the WT (=1). For (*D* and *E*), the effects of the mutations on expression and terminal leaflet margin complexity were tested for significance using a mixed-effects model (*P < 0.05, **P < 0.01, ***P < 0.001). WT: n = 7, *mut1*: n = 3, *mut2*: n = 7, *mut3*: n = 6, *mut4*: n = 6, *rco^enh−^*: n = 7, *mut5*: n = 3, *mut6*: n = 3, *mut7*: n = 3, *rco^−^*: n = 3. (*F*) Silhouettes of representative leaf 5 of the mutants shown in (*C*). Black arrowheads indicate the dissection of the terminal leaflet in the *RCO* gain-of-function mutants, while red arrowheads indicate stipule outgrowths. (Scale bar: 1 cm.) (*G*) Photographs of representative lateral leaflets (first pair) of leaf 5 from WT, *mut4*, *rco^enh-^*, and *mut7*. (Scale bar: 1 cm.) (*H*) Boxplots indicating petiolule narrowness (see *Materials and Methods* details “Leaf phenotype analysis”) of the alleles shown in (*G*). The effects of the mutations on petiolule narrowness were tested for significance using a linear model (*P < 0.05, **P < 0.01, ***P < 0.001). WT: n = 22, *mut4*: n = 22, *rco^enh-^*: n = 36, *mut7*: n = 14. Boxplots (*D*, *E*, and *H*): boxes display the interquartile range (25th and 75th percentiles). Thicker line: median.

Leaves provide an attractive system for understanding mechanisms of regulatory evolution because their shapes present considerable heritable variation, underpinned by changes in expression of developmental genes. Leaf form can be described as simple (if the leaf blade is undivided) or dissected (if the blade is divided into distinct leaflets) (14–16). Additionally, leaves can show intermediate complexity and elaborate outgrowths that are less pronounced than leaflets, referred to as lobes. It was previously shown that regulatory diversification after tandem gene duplication of the *REDUCED COMPLEXITY* (*RCO*) homeobox gene (Fig. 1*A*) underlies the emergence of complex (dissected and lobed) leaf shapes in the Brassicaceae (17, 18). This process involved the acquisition of an *RCO* gene expression domain comprising distinct expression foci at the leaf base, which contrasts with the distal expression domain of its ancestral paralog *LATE MERISTEM IDENTITY 1* (*LMI1*) in the leaf margin and vestigial appendages called stipules (17).

The shift in gene expression from an *LMI1* to an *RCO* pattern played a key role in crucifer leaf shape evolution. Transgenic experiments indicated that an approximately 500 bp enhancer element, *RCOenh^500^*, was sufficient to control this shift in gene expression domain, but causal sequence changes and mechanisms remain unknown (19). It is also unclear to what degree transgenic assays using reporter genes reflect the function of regulatory elements in their native genomic context—an issue that can potentially be resolved by studying CRISPR/Cas9 regulatory alleles. Leaves of *rco* loss-of-function alleles show reduced complexity since the RCO protein is a growth repressor that acts to separate distinct leaflets to form complex leaves (17, 20). Conversely, since LMI1 normally acts to repress stipule growth, loss of *LMI1* function causes the conversion of vestigial stipules to ectopic leaves at the leaf base [Fig. 1*B* and Vuolo et al. (21)]. These phenotypes can therefore be used to evaluate the consequences of regulatory mutations of *RCO* and *LMI1* and the mechanistic basis for how the distinct gene expression domain conferred by *RCOenh^500^* evolved from its ancestral *LMI1* counterpart.

Here, we used CRISPR/Cas9 genome editing to mutagenize the *RCO* and *LMI1* enhancers in *Cardamine hirsuta*. We assayed phenotypes and transcript levels of 17 regulatory alleles of the two enhancers, including chimeric *LMI1/RCO* enhancer alleles. We found that the more recently evolved *RCO* enhancer was subject to more negative regulation and, while the *LMI1* enhancer was strictly required for *LMI1* gene expression, *RCO* enhancer deletion caused only partial loss of function. We used reporter assays of these enhancer variants to demonstrate how a repressive sequence in the *RCO* enhancer delimits the *RCO* expression domain and how a second sequence contributes to activating *RCO* expression. The repressive sequence that shapes the distinct *RCO* expression domain at the leaf base arose in association with a local within-enhancer duplication. Our findings underscore the significance of gene repression in evolving a gene expression domain and show that repressive sequences underlying this effect can arise through sequence duplication. Our results also demonstrate how combining dense CRISPR/Cas9 mutagenesis with reporter gene assays is a powerful strategy to identify enhancer regulatory features that underlie morphological evolution.

## Results and Discussion

### Generation of Mutant Alleles in *RCO* and *LMI1* Enhancers Using CRISPR/Cas9.

**Fig. 2.**
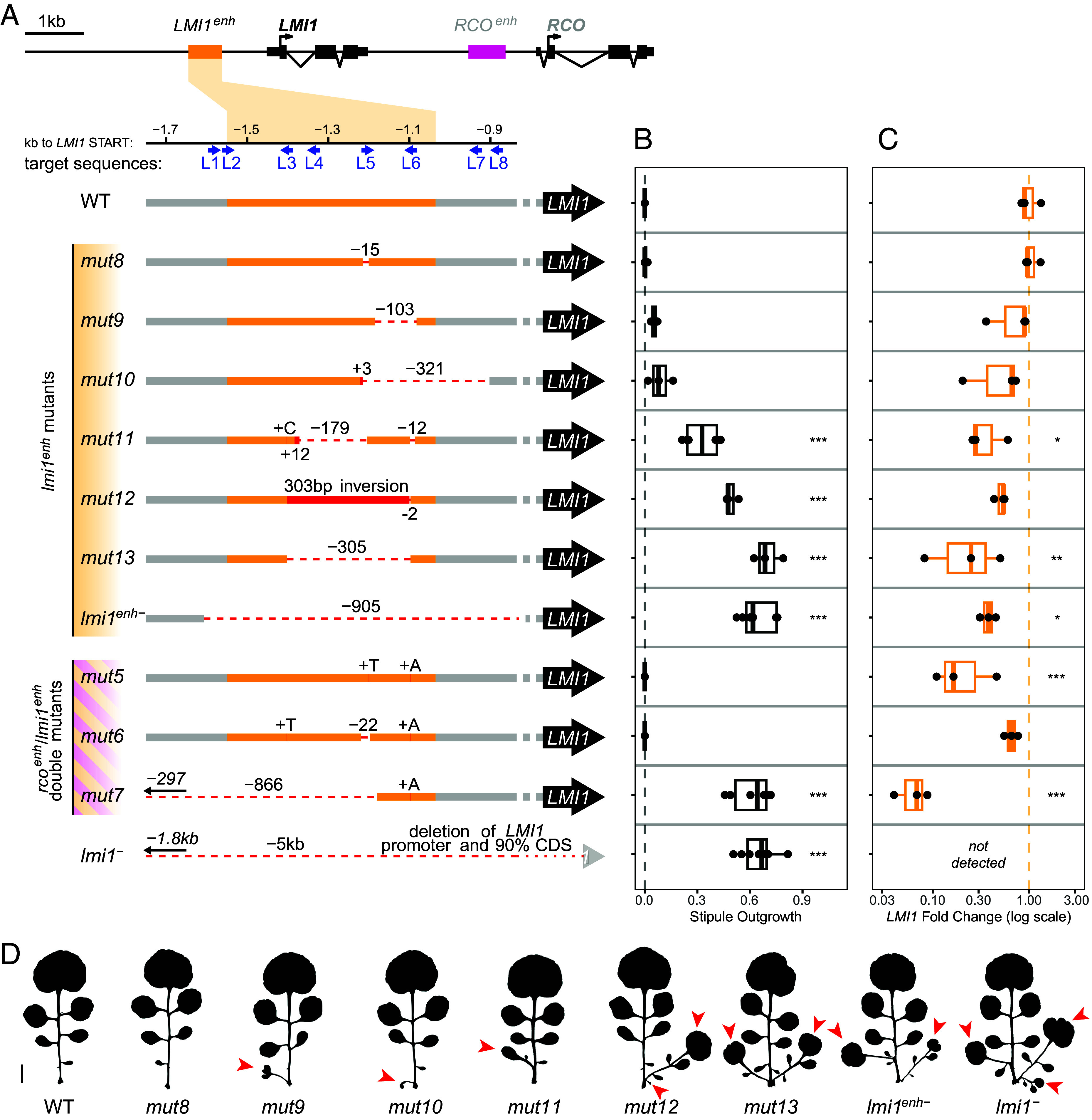
Mutations in the *LMI1* enhancer readily cause strong *LMI1* loss-of-function phenotypes. (*A*) Schematic representation of the CRISPR-Cas9-generated mutant alleles in the *LMI1* enhancer. Blue arrows show the position and direction of the sgRNA target sequences used. The first seven alleles indicate *lmi1^enh^* mutants, and the remaining three indicate *rco^enh^*/*lmi1^enh^* double mutants. (*B*) Stipule Outgrowth Index (see *Materials and Methods* details “Leaf phenotype analysis”) of the mutants shown in (*A*). Increasing values indicate longer and more frequent stipule outgrowths. The dashed line indicates the absence of stipule outgrowth (=0). The effects of the mutations on stipule growth were tested for significance using a mixed-effects model (*P < 0.05, **P < 0.01, ***P < 0.001). WT: n = 3, *mut8*: n = 3, *mut9*: n = 3, *mut10*: n = 3, *mut11*: n = 6, *mut12*: n = 3, *mut13*: n = 3, *lmi1^enh-^*: n = 7, *mut5*: n = 3, *mut6*: n = 3, *mut7*: n = 6, *lmi1^−^*: n = 8. (*C*) Relative *LMI1* expression levels of the alleles shown in (*A*) as determined by qPCR, measured in 14-d-old seedling apices. The dashed line indicates expression in the WT. The effects of the mutations on expression were tested for significance using a linear model (*P < 0.05, **P < 0.01, ***P < 0.001). N = 3 biological replicates for all the samples. Boxplots in (*B* and *C*): boxes display the interquartile range (25th and 75th percentiles). Thicker line: median. (*D*) Silhouettes of a representative leaf 5 of the mutants shown in (*A*). Red arrowheads indicate stipule outgrowths. (Scale bar: 1 cm.)

**Fig. 3.**
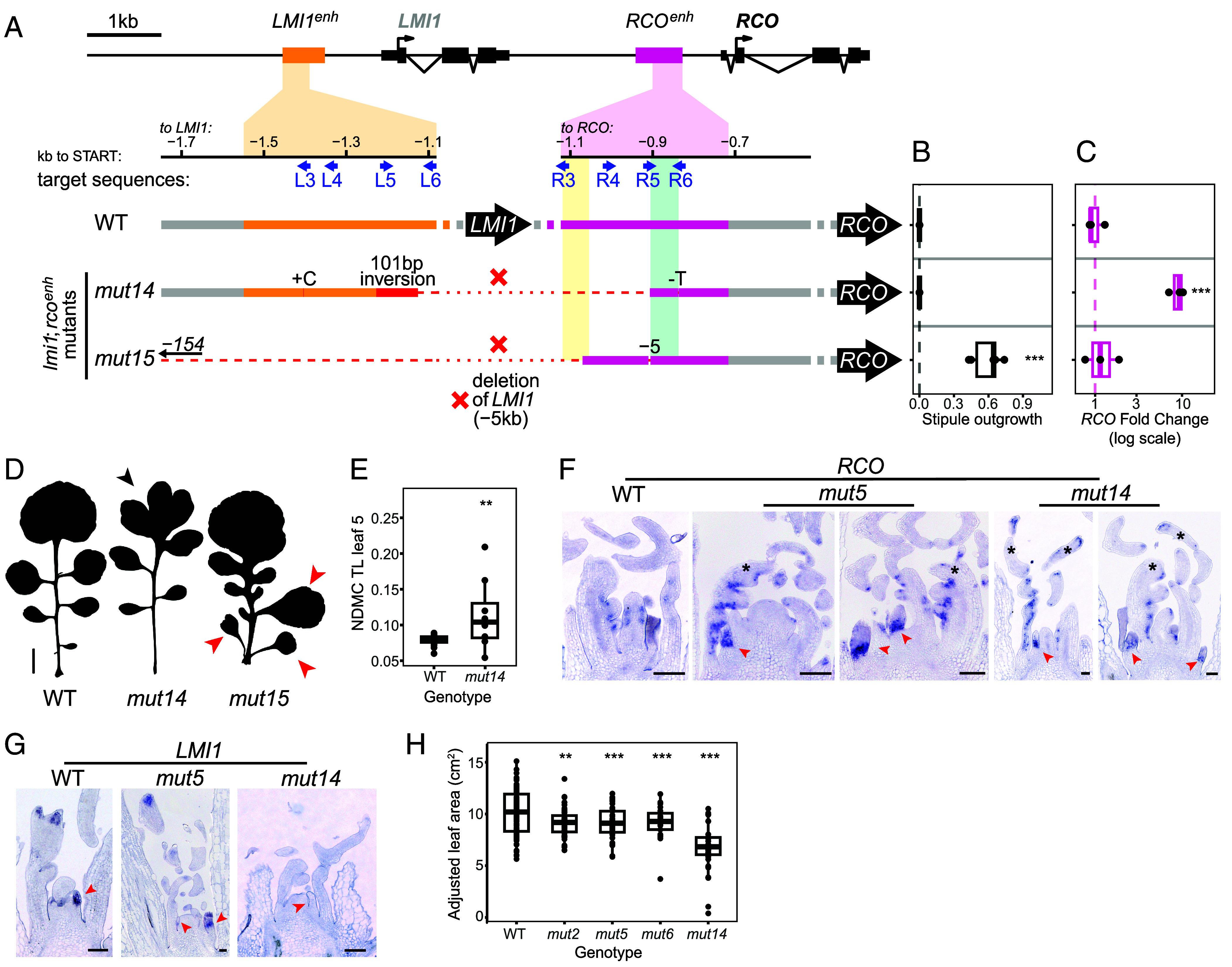
Effect of CRISPR-Cas9-generated hybrid *LMI1/RCO* enhancers on leaf morphology and *RCO* expression. (*A*) Schematic representation of the two CRISPR-Cas9-generated mutant alleles, where targeting both the *LMI1* and the *RCO* enhancer resulted in an *LMI1* gene deletion. Yellow- and green-shaded boxes in *RCO^enh^* indicate the same regions as in Fig. 1*C*. (*B*) Stipule Outgrowth Index (see *Materials and Methods* details “Leaf phenotype analysis”) of the mutants shown in (*A*). Increasing values indicate longer and more frequent stipule outgrowths. The dashed line indicates absence of stipule outgrowth (=0). The effects of the mutations on stipule growth were tested for significance using a mixed-effects model (*P < 0.05, **P < 0.01, ***P < 0.001). N = 6 for all the samples. (*C*) Relative *RCO* expression levels of the alleles reported in (*A*) as shown by qPCR, measured in 14-d-old seedling apices. The dashed line indicates control expression in the WT. The effects of the mutations on expression were tested for significance using a linear model (*P < 0.05, **P < 0.01, ***P < 0.001). N = 3 biological replicates for all the samples. (*D*) Silhouettes of a representative leaf 5 of the mutants shown in (*A*). The black arrowhead indicates the dissection of the terminal leaflet in the *RCO* gain-of-function mutant, while red arrowheads indicate stipule outgrowths. (Scale bar: 1 cm.) (*E*) NDMC of the terminal leaflet of leaf 5 from WT and *mut14*. The dashed line indicates the WT. The effects of the mutation on terminal leaflet margin complexity were tested for significance using a mixed-effects model (**P < 0.01). WT: n = 25, *mut14*: n = 8. (*F* and *G*) Representative in situ hybridization micrographs of *RCO* (*F*) and *LMI1* (*G*) transcripts on vegetative meristem sections of WT, *mut5,* and *mut14* plants. Black asterisks indicate ectopic expression in the TLs, while red arrowheads show stipules. Samples observed by genotype and gene: *LMI1* probe on WT = 5, *LMI1* probe on *mut5* = 1, *LMI1* probe on *mut14* = 2, *RCO* probe on WT = 4, *RCO* probe on *mut5* = 13, *RCO* probe on *mut14* = 3. (Scale bar: 100 μm.) (*H*) Adjusted leaf area values (see *Materials and Methods* details “Normalized leaf area for node effects”) of leaves from nodes 5, 6, 7, and 8 of WT and the alleles showing an RCO gain-of-function phenotype (*mut2*, *mut5*, *mut6*, and *mut14*) which overall show reduced leaf surface. The effects of the mutations on leaf area were tested for significance using a mixed-effects model (**P < 0.01, ***P < 0.001). WT: n = 92, *mut2*: n = 48, *mut5*: n = 41, *mut6*: n = 21, and *mut14*: n = 32. Boxplots in (*B*, *C*, *E*, and *H*): boxes display the interquartile range (25th and 75th percentiles). Thicker line: median.

To investigate the function of *RCO* and *LMI1* enhancers in their native genomic context, we generated CRISPR/Cas9 alleles using eight single guide RNAs for each enhancer. In this way, we isolated five mutants in the *RCOenh^500^* (Fig. 1*C* and *SI Appendix*, Fig. S1), seven mutants in the *LMI1enh^500^* (Fig. 2*A* and *SI Appendix*, Fig. S3) and three carrying mutations in both enhancers (Figs. 1*C* and 2*A* and *SI Appendix*, Fig. S1). Additionally, we recovered two complex alleles (*mut14* and *mut15*) where the *LMI1* gene is deleted, resulting in the fusion of *LMI1* upstream sequences to the *RCOenh^500^*, with one of them (*mut14*) creating a hybrid *LMI1enh^500^*/*RCOenh^500^* (Fig. 3*A* and *SI Appendix*, Fig. S6). We subsequently investigated the leaf phenotypes of these alleles as well as their impact on *LMI1* or *RCO* transcript levels using qRT-PCR (Figs. 1 *D* and *E*, 2 *B* and *C*, and 3 *B* and *C*), with the aim of understanding the functional properties of these enhancers and the regulatory logic underlying their diversification following gene duplication.

### Characterization of Mutant Alleles in *RCOenh^500^*.

We characterized eight *rco^enh^* mutant alleles, three of which also carried mutations in *LMI1enh^500^* (Fig. 1, *SI Appendix*, Figs. S1 and S2, and Dataset S1). Among these eight, one (*rco^enh-^*) was a deletion of 755-bp that spans the entire enhancer sequence (Fig. 1*C*). This deletion allele resulted in a 50% reduction in transcript levels and a leaf phenotype where leaflets are incompletely separated from the rachis and instead form deep lobes (Fig. 1 *D*–*H* and *SI Appendix*, Fig. S1). This phenotype is much weaker than the *rco* reference allele, which is caused by a premature stop codon (17). Therefore, other regulatory sequences must act redundantly with *RCOenh^500^* to promote *RCO* gene expression, as has been shown in other systems (22, 23). Two other alleles (*mut4* and the double mutant *mut7*) yielded very weak loss-of-function phenotypes, where petiolules do not form properly, resulting in weaker leaflet separation defects than in the *rco^enh-^* mutant (Fig. 1 *G* and *H* and *SI Appendix*, Figs. S1 and S2). No reduction in *RCO* transcript was detected in these two mutants by qRT-PCR (Fig. 1*E*). Three additional alleles (*mut2*, *mut5*, and *mut6*) had a conspicuous leaf phenotype showing increased dissection of the terminal leaflet (TL) and an increase in *RCO* transcript levels (Fig. 1 *D*–*F*, black arrowheads in F showing the TL dissection). This TL phenotype resembles transgenic lines where *RCO* is ectopically expressed in the terminal leaflet (20), suggesting that these are gain-of-function alleles, where elevated *RCO* expression causes increased terminal leaflet complexity. Notably, *mut6* is a hybrid of sequences from the two enhancers with 124 bp from *LMI1enh^500^* inserted into *RCOenh^500^* (Fig. 1*C*), raising the possibility that this 124 bp sequence contributes to the elevated *RCO* expression in this mutant. Finally, one allele (*mut3*) showed increased *RCO* transcript levels and a tendency to increase TL complexity that was not statistically significant (Fig. 1 *C*–*F*). Overall, these results highlight two properties of the *RCOenh^500^*: First, it is partially, but not fully required for *RCO* gene expression, as an enhancer deletion allele yielded a mild phenotype and it reduced, but did not abolish gene expression. Second, it contains repressive sequences that limit *RCO* gene expression and negatively regulate leaf dissection. Mutations in such repressive sequences appear easier to recover than strong loss-of-function alleles in this experimental design.

### Characterization of Mutant Alleles in *LMI1enh^500^*.

To understand the profile of *LMI1enh^500^* response to mutation, we characterized ten *LMI1enh^500^* alleles, three of which were double *LMI1enh^500^*/*RCOenh^500^* mutants (Fig. 2, *SI Appendix*, Figs. S3–S5, and Dataset S2). The *LMI1enh^500^* profile was very different to what we observed for *RCOenh^500^*. A 900-bp deletion allele encompassing the entire enhancer (*lmi1^enh−^*) reduced *LMI1* transcript levels and gave a similarly strong phenotype to the *lmi1^−^* deletion allele generated for the purpose of this study (Fig. 2 *A*–*D*, *SI Appendix*, Figs. S3 and S4, and Dataset S3). In both alleles, vestigial stipules were fully converted to leaves (Fig. 2*D*). Four additional alleles resulted in reduced *LMI1* transcript levels and strong *lmi1* loss-of-function phenotypes (*mut11*, *mut12*, *mut13*, and *mut7* which also carries a mutation in *RCO*, Fig. 2 *A*–*D*). These results indicate that the mutated regions contain sequences necessary for *LMI1* expression and that the *LMI1* enhancer sequence is more prone to resulting in loss of function upon mutation than its *RCO* counterpart. Additionally, our analysis of all seventeen *rco^enh^* and *lmi1^enh^* mutant alleles (Figs. 1 and 2) indicates that the *RCO* enhancer is more prone to generate gain- rather than loss-of-function alleles upon mutagenesis, consistent with it having evolved more negative regulation than the ancestral *LMI1* enhancer.

### Characterization of Complex Alleles with Concurrent *LMI1enh^500^* and *RCOenh^500^* Deletions.

Next, we studied the two complex alleles arising from concurrent mutation of *LMI1enh^500^* and *RCOenh^500^* (Fig. 3 *A*–*D*, *SI Appendix*, Fig. S6, and Dataset S4). The first allele, *mut14*, arose through deletion of the *LMI1* gene which resulted in a fusion of the *LMI1* and *RCO* enhancers (400-bp of the 5′ of *LMI1enh^500^*, 101 of which are inverted, and 200-bp of the 3′ of *RCOenh^500^*, Fig. 3*A*). This mutant showed increased expression of *RCO* (Fig. 3*C*) accompanied by an increased TL dissection phenotype (Fig. 3 *D* and *E*), indicating that it is an *RCO* gain-of-function allele. The second allele, *mut15*, had a larger deletion of *LMI1* (5.4-kb) compared to *mut14*, removing the entire *LMI1enh^500^* and 350-bp upstream sequence (Fig. 3*A*). The leaf phenotype of *mut15* showed both *RCO* (Fig. 3*D*) and *LMI1* loss-of-function phenotypes (Fig. 3*B* and *SI Appendix*, Fig. S6), consistent with the large deletion eliminating the function of both genes. The fact that *mut15*, but not *mut14*, showed an *RCO* loss-of-function phenotype (Fig. 3*D*) indicates that the deleted *RCO* sequences contain information required for *RCO* expression but can be compensated by *LMI1* upstream sequences in *mut14* that are removed in *mut15* (Fig. 3*A*) and, in combination with the *RCO* upstream sequence still present in *mut14*, can activate gene expression in the *RCO* domain. Strikingly, *mut14* did not show an *lmi1* loss-of-function phenotype despite deletion of the entire *LMI1* gene (Fig. 3*B* and *SI Appendix*, Fig. S6). This observation indicates that the hybrid enhancer and regulatory sequence created in this deletion allele can drive *RCO* expression in a way that allows it to functionally substitute for *LMI1*. This is consistent with previous findings that *LMI1* can complement the *rco* loss-of-function phenotype when expressed as a transgene in the *RCO* domain (17) and provides genetic evidence from editing in the native genomic context that differences in expression patterns of *LMI1* and *RCO* genes underlie their different roles in development. Consistent with these ideas, in situ hybridization of *RCO* in *mut14* and the previously described gain-of-function *mut5* allele (Fig. 1*D*) showed ectopic *RCO* expression in stipules, which is not detectable in the wild type (Fig. 3*F*, red arrowheads on stipules and *SI Appendix*, Fig. S7). *LMI1* transcripts could be detected in *mut5*, but not in *mut14*, consistent with the deletion of *LMI1* in the *mut14* allele (Fig. 3*G*). Additionally, *RCO* transcripts were detected in the terminal leaflets of *mut14* and *mut5* leaves, consistent with the terminal leaflet lobing phenotype in these alleles (Fig. 3*F*). Notably, all the alleles with increased *RCO* expression and higher terminal leaflet complexity (*mut2*, *mut5*, *mut6*, and *mut14*) had smaller leaves (Fig. 3*H*), indicating that elevated *RCO* expression causes pleiotropic effects on leaf development.

### Transgenic Validation of Regulatory Sequences within *RCOenh^500^*.

**Fig. 4.**
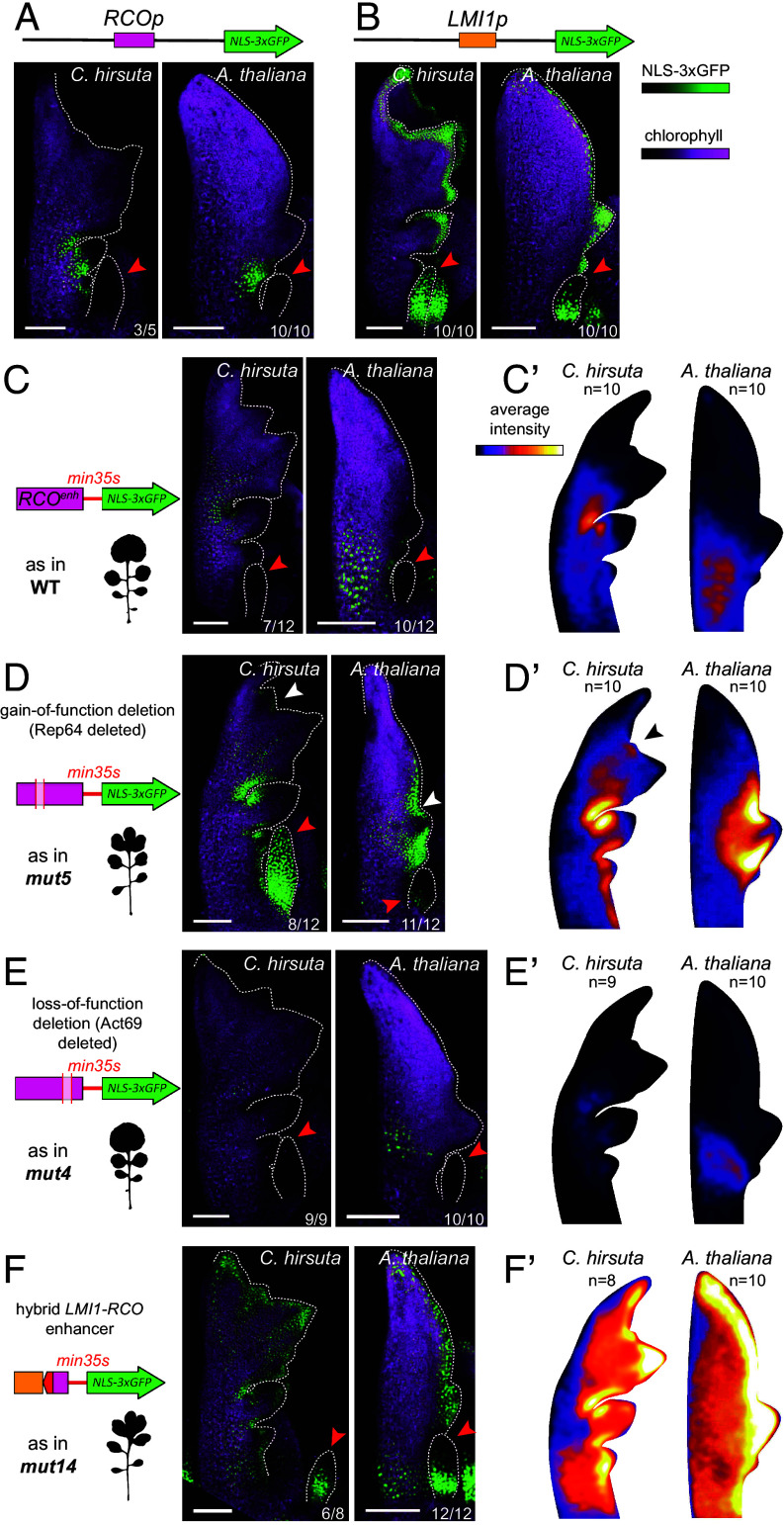
Reporter gene validation of effects of regions Rep64 and Act69 identified through CRISPR indicates their modular function. (*A* and *B*) Expression of *ChRCOp:NLS-3xGFP* (*A*) and *ChLMI1p:NLS-3xGFP* (*B*) in *C. hirsuta* and *Arabidopsis thaliana* leaf primordia. (*C*) Expression conferred by the WT *RCO^enh^* sequence (631 bp) when placed upstream a cassette comprising the minimal *35 s* promoter and *NLS-3xGFP* in *C. hirsuta* and *A. thaliana* leaf primordia. (*C’*) Normalized leaf primordium expression profile of the construct in (*C*) averaged from 10 independent transformants. (*D*) Expression conferred by a modified *RCO^enh^* sequence containing the 64bp g.o.f. deletion (Rep64, as in *mut5*; 567 bp) upstream the same *min35s:NLS-3xGFP* cassette in *C. hirsuta* and *A. thaliana* leaf primordia. (*D’*) Normalized leaf primordium expression profile of the construct in (*D*) averaged from 10 independent transformants. (*E*) Expression conferred by a modified *RCO^enh^* sequence containing the 69bp l.o.f. deletion (Act69, as in *mut4*; 562 bp) when placed upstream of the same *min35s:NLS-3xGFP* cassette in *C. hirsuta* and *A. thaliana* leaf primordia. (*E’*) Normalized leaf primordium expression profile of the construct in (*E*) averaged from 9 and 10 independent transformants for *C. hirsuta* and *A. thaliana*, respectively. (*F*) Expression conferred by the hybrid *LMI1-RCO* enhancer from *mut14* (613 bp) when placed upstream of the same *min35s:NLS-3xGFP* cassette in *C. hirsuta* and *A. thaliana* leaf primordia. (*F’*) Normalized leaf primordium expression profile of the construct in (*F*) averaged from 8 and 10 independent transformants for *C. hirsuta* and *A. thaliana,* respectively. (*A*–*F*): Red arrowheads show the stipules. The white arrowheads in (*D*) indicate the fluorescence detected in the TL of *C. hirsuta* and in the upper sinus of *A. thaliana.* The black arrowhead in (*D’*) indicates ectopic expression in the *C. hirsuta* terminal leaflet. (Scale bar: 100 μm.) The number of independent transgenic lines showing the reported expression pattern is shown at the bottom right-hand side of the confocal micrographs.

To perform a fine dissection of the *RCOenh^500^*, we compared the eight *rco^enh^* alleles to identify specific sequences that potentially underlie repression or activation of gene expression and evolution of the novel *RCO* gene expression domain. For example, *mut5* harbors a deletion of 64-bp, which defines the smallest lesion that results in increased *RCO* transcript levels and a gain-of-function phenotype, and overlaps with the 232-bp deletion in *mut2* that yields a similar increase in *RCO* transcript and leaf complexity (Fig. 1 *C*–*F*, region highlighted in yellow in C). Therefore, this 64 bp sequence likely contains negative regulatory elements that delimit the correct *RCO* expression domain. Comparing *mut7* and *mut4*, which both yield loss-of-function phenotypes (Fig. 1 *G* and *H*), identified a shared 69-bp sequence (region between R5 and R6, highlighted in green in Fig. 1*C*), which likely contains positive regulatory elements for *RCO*. To test these predictions, we used reporter gene assays to evaluate whether these *rco^enh^* sequence variants are in themselves sufficient to alter gene expression in the manner indicated by the CRISPR/Cas9 alleles. Specifically, we recreated the two candidate deletions causing *RCO* gain- and loss-of-function; first, the 64-bp deletion in *mut5* that we named Rep64 for “Repressor 64” (the yellow bar in Fig. 1*C*) and second, the 69-bp deletion in *mut4* that we named Act69 for “Activator 69” (the green bar in Fig. 1*C*). In these assays, we also recreated the hybrid *RCO*/*LMI1* enhancer in *mut14* to test whether this hybrid enhancer is sufficient to cause broadened or elevated *RCO* gene expression. We subsequently compared the gene expression driven by each mutant enhancer to the wild-type *RCOenh^500^* (Fig. 4 *A*–*F’*). These are important experiments to further understand the functional significance of CRISPR/Cas9-defined sequences. For example, confirmation that the activation or repression conferred by these mutated *RCOenh^500^*sequences can act in isolation would indicate that they function as distinct modules operating, at least to some degree, independently of neighboring genomic sequences.

In these assays, the wild-type enhancer fragment drives gene expression in a domain faithful to a full reporter gene for *RCO* expression when positioned 5′ to a minimal *35S* promoter (Fig. 4 *A* and *C*), as reported previously (19). In addition to imaging individual transgenic lines, we also estimated the average expression of each genotype to account for variation between independent transgenic lines (Fig. 4 *C’–F’* and *SI Appendix*, Fig. S8). Overall, we observed expression patterns consistent with the corresponding CRISPR/Cas9 alleles (Fig. 4 *C*–*F*). Specifically, constructs carrying the *mut5* deletion or the hybrid *mut14* enhancer resulted in increased and broadened gene expression in distal parts of the leaf and in stipules where the wild-type *RCOenh^500^* does not drive gene expression (Fig. 4 *A*, *D*, and *F*). Conversely, the *mut4* deletion construct resulted in reduced expression relative to the wild-type *RCOenh^500^* expression (Fig. 4 *A* and *E*). Thus, we conclude that Rep64 harbors negative regulatory elements, while Act69 contains elements for positive regulation (*SI Appendix*, Fig. S9 and Table S11). Each of these sequences acts as a distinct module to define the spatial pattern of *RCO* expression. Notably, the Rep64 deletion in *mut5* removes half of a conserved 50-bp sequence duplication which distinguishes *RCOenh^500^* orthologs from their *LMI1enh^500^* counterparts (*SI Appendix*, Fig. S9 and Dataset S5). By replacing the duplicated sequence within *RCOenh^500^* with its counterpart sequence from *LMI1 enh^500^*, which lacks the duplication, we observed ectopic gene expression in distal parts of the leaf and in stipules (*SI Appendix*, Fig. S10*A*). Therefore, this duplication within *RCOenh^500^* contributes to delimiting the *RCO* expression domain via negative regulation. Finally, we created a construct that mirrors the CRISPR *RCOenh^500^* enhancer deletion by deleting the *RCOenh^500^* sequence in the context of the full *RCO* reporter gene (*SI Appendix*, Fig. S10*B*). We observed that this deletion almost completely abolishes GFP expression (*SI Appendix*, Fig. S10*B*). This does not match the reduced, rather than absent, *RCO* expression observed in the *RCOenh^500^* enhancer deletion allele (Fig. 1*E*). This finding indicates that sequences acting redundantly with *RCOenh^500^* are unlikely to reside in the 3.2 kb sequence used in the *RCO* reporter gene, or alternatively, if they reside there, they can only function in the native genomic context. In summary, our findings highlight the importance of combining CRISPR/Cas9 mutagenesis and reporter gene assays to obtain fine-grained information on enhancer function during development and evolution.

## Conclusions

By comparing the genetic profile of mutations in an ancestral and derived enhancer, we provide insights into the genetic logic underlying enhancer sequence evolution, gene expression changes, and morphological diversity. The derived *RCO* enhancer has two main differences relative to its ancestral *LMI1* counterpart. First, it is partially rather than strictly required for gene expression, resulting in *RCO* expression being better buffered to *enh^500^* mutations. This feature may have helped to stabilize the novel *RCO* expression domain following gene duplication from *LMI1*, similar to previous findings (24). Second, and more importantly, *RCOenh^500^* is subject to more negative regulation than *LMI1enh^500^*. When this negative regulation is lost, for example, in *mut5* and *mut14* deletion alleles, *RCO* is expressed in more distal parts of the leaf and in stipules, thus resembling the ancestral gene expression pattern of *LMI1*. We propose that this negative regulation within *RCOenh^500^* was a pivotal mechanism to evolve the restricted *RCO* expression domain at the leaf base, which underpinned the evolution of complex leaf forms in crucifers (17, 20).

Our approach allowed us to link genotypes generated by genome editing to morphological phenotypes by considering corresponding changes in gene expression—a connection often missing in the field. For some alleles, the correspondence between phenotypic, qRT-PCR, and reporter gene data was incomplete. Several reasons could account for this, including the inability of qRT-PCR to capture cell- and stage-specific gene expression information, position effects in transgenic reporter gene assays, and the influence of regulatory sequences not included in reporter genes (for details, see *SI Appendix, Supplemental Text 1*). The development of efficient transgenic knock-in strategies in plants for allele-specific expression analysis in the native genomic context using fluorescent tags will be an important next step toward addressing these issues.

Sequence duplications at two levels likely contributed to the evolution of leaf complexity by *RCO*. First, duplication of the ancestral *LMI1* gene including its regulatory sequences created the potential to evolve the new *RCO* gene expression domain. Second, a smaller duplication of a specific sequence within *RCOenh^500^* accompanied and may have facilitated, the evolution of negative regulatory elements which helped delimit the novel *RCO* expression domain. Identifying specific transcription factors that bind sites (TFBS) in Rep64 and mediate these repressive effects, together with determining the evolutionary history of these TFBS sequences, will be an important follow-up to this study. In addition, the evolution of repressive sequences within the *RCOenh^500^* may have limited the potential pleiotropic effects caused by novel expression of *RCO,* a potent growth repressor, since *rco^enh^* alleles where these sequences are deleted (*mut2, mut5, mut6, mut14*) show reduced leaf size and altered leaf shape. Thus, our results demonstrate the importance of repressive sequences in shaping the gene expression output of enhancers and curtailing potentially pleiotropic effects of developmental genes during morphological evolution.

## Materials and Methods

Full description of the materials used and protocol-level methods are shown in the *SI Appendix*, including: Experimental model and plant growth conditions; Cloning and generation of transgenic plants and mutants; Genotyping; Quantitative RT PCR; Sample preparation and imaging; Image registration and analysis; In situ hybridization assays; Leaf phenotype analysis; Bioinformatics, quantification; and statistical analyses.

## Supplementary Material

Appendix 01 (PDF)

Dataset S01 (TXT)

Dataset S02 (TXT)

Dataset S03 (TXT)

Dataset S04 (TXT)

Dataset S05 (TXT)

## Data Availability

FASTA alignment files (plain-text .txt) for the sequences discussed are provided as follows: Dataset S1 (*rco^enh^* mutants), Dataset S2 (*lmi1^enh^* mutants), Dataset S3 (*lmi1^−^* mutant), Dataset S4 (*mut14* and *mut15*), and Dataset S5 (Alignment of *RCO* enhancer and *LMI1* enhancer sequences). The sliding window script and Dataset S5 have been uploaded to a public repository (25). All additional details are in the supporting information.
