# Supplementary material for: Evolution of repressive sequences within an enhancer contributed to morphological diversity in crucifer plants
Source: Proc Natl Acad Sci U S A. 2025 Dec 16;122(51):e2515732122. doi: 10.1073/pnas.2515732122 (PMC12745806; doi:10.1073/pnas.2515732122)
Supplement: Supplementary file 1 — Appendix 01 (PDF) [file pnas.2515732122.sapp.pdf]

PNAS

Supporting Information for
Evolution of repressive sequences within an enhancer contributed to morphological diversity in crucifer plants.

Alessandro Popoli[1,2], Remco A. Mentink[1,3], Lisa Brombach[1], Nora Papadima-Karanikou[1], Manuel Buendia-Monreal[1,4], Mingming Fang[1], Manas Joshi[1,5], Saiko Yoshida[1,6], Stefan Laurent[1,7], Peter Huijser[1], Miltos Tsiantis[1*]

*Miltos Tsiantis.
Email: tsiantis@mpipz.mpg.de

This PDF file includes:

Supporting text – extended Methods section
Figures S1 to S10
Tables S1 to S11
Supplementary Text 1
Dataset descriptions S1 to S5
SI References

Supporting Information Text

Experimental Model and Plant growth conditions

All mutants used in this study were generated in *Cardamine hirsuta* Oxford (*Ch* Ox WT) background (1). The transgenic lines were generated in *C. hirsuta* Ox background and *Arabidopsis thaliana* Col-0 (*At*) background. Seeds were stratified directly on soil for a minimum of one day at 4°C in darkness. Subsequently, unless otherwise stated, plants were cultivated in soil under long-day conditions (16 hours of light, 8 hours of darkness) in greenhouses. The light intensity at bench level ranged from approximately 70 to 110 $\mu mol\, m^{-2}\, s^{-1}$, and temperatures were maintained at 22°C during the day and 20°C overnight.

Cloning and generation of transgenic plants and mutants

Cloning of the vectors used in this study

The 20bp-target sequences used to target *LMI1enh*^{500} (L1, L2, L3, L4, L5, L6, L7, and L8, Table S1) and *RCOenh*^{500} (R1, R2, R3, R4, R5, R6, R7, and R8, Table S1) were designed manually, blasting the fragments on the *C. hirsuta* genome v1.0 to avoid sequences likely to act off-target. The expression cassettes with 2 single-guide RNAs (sgRNAs) each were designed according to Yan et al.(2). Synthesis and cloning into pUC57 (Amp) was performed by the company GenScript (USA). Cassettes containing L4-L5 and L3-L6 were cloned into pDE-Cas9 (a kind gift from Holger Puchta)(3) through LR reaction (Thermo Fisher) to generate pDE-Cas9_L4-L5, used to generate *mut8*, and pDE-Cas9_L3-L6, used to generate *mut12* and *mut13*. To create higher-order sgRNAs expression cassettes, single 2-sgRNAs cassettes were combined together using *Kpn*I, *Spe*I, and *Xba*I (NEB), exploiting the sticky ends produced by *Spe*I and *Xba*I. In this way, we generated pDE-Cas9_R3-R4-R5-R6, used to generate *mut1*, *mut2*, *mut3*, and *mut4*, pDE-Cas9_R1-R2-R7-R8, used to generate *rco*$^{enh-}$, pDE-Cas9_L3-L4-L5-L6, used to generate *mut9*, *mut10*, and *mut11*, pDE-Cas9_L1-L2-L7-L8, used to generate *lmi1*$^{enh-}$, and pDE-Cas9_L3-L4-L5-L6-R3-R4-R5-R6, used to generate *mut5*, *mut6*, *mut7*, *mut14*, and *mut15*.

For generating a null *LMI1* mutant allele, we used the CRISPR/Cas9 online target predictor CCTop (4, 5). Target sequences LN1, LN2, LN3, and LN4 (Table S1), which target 3.5 kb upstream *LMI1* cds and at the 3'-end of *LMI1* cds, have been consequently chosen among the most favorable hits from the CCTop analysis. Synthesis and subcloning in pUC57 (Amp) of the 4 sgRNAs containing these target sequences into a single cassette was performed by the company GenScript. As before, this cassette was cloned into pDE-Cas9 through LR reaction to generate pDE-Cas9_LN1-LN2-LN3-LN4, used to generate *lmi1*$^{-}$, a 5-kb-deletion mutant of the *LMI1* gene that includes 3.7 kb upstream the *LMI1* start codon.

Table S1. Target sequences used for CRISPR/Cas9 in this study.

**Name**	**Target sequence**
L1	AAGTTAATAAAAGAATGTGG
L2	AGAAGAGTACACAGTAGATG
L3	ACCTGTAAGAGTATAGGGTG
L4	GTGAAAAATTAATATGCAAA
L5	GAAGGCATAACCAAAGGAGA
L6	ATTTCATGTGTTAATATAGG
L7	TTTGACGGCTCTTCTAAACG
L8	ATATAGTATAGACCAATAAA
R1	AAACAATTGCAGATATCTAT
R2	TTTCGCCTGTCCACTTGATA
R3	GTATATGTGCAGTACCTGAA
R4	AAAATTTGTGCAGTTTTAGA
R5	CCCTGTTGTCAGTTATTCTA
R6	TTCATGTAATTAATATAGGT
R7	TCAGAATTTTCTTTTCAACT
R8	TGAAATTCAGTCGATTAAGG
LN1	CTCACAATAATTATATAATG

LN2	AAAGCCTCCTCTTTCTATGG
LN3	CACGGCGGAGATTCCATCGG
LN4	ACCACCAATCAAATCACAGG

To generate *pChLMI1::LMI1cds:Venus* and *pChRCO::RCOg:Venus*, the expression cassettes were created similarly to Hajheidari et al. (6), with *pChLMI1::LMI1cds:Venus* and *pChRCO::RCOg:Venus* followed by the octopine synthase (OCS) terminator. These cassettes were cloned into the binary vector pMLBART with *Not*I (NEB) for Agrobacterium transformation. *pChRCO::RCOg:Venus* is also described in Wang et al. (7). In the case of *pChLMI1::NLS:3xGFP*, *pChRCO::NLS:3xGFP*, and *pChrco*$^{enh-}$*::NLS:3xGFP*, the *ChLMI1* promoter was amplified from genomic DNA of an *A. thaliana* Col-0 plant transformed with *pChLMI1::GUS,* while the *ChRCO* promoter was amplified from *pChRCO::RCOg:Venus*. For the *ChLMI1* promoter, we used the sequence from -1 to -3588 bp upstream the *LMI1* translation start codon (ATG); for the *ChRCO* promoter, we used the sequence from -1 to -3198 bp upstream the *RCO* ATG. The amplicons were subsequently cloned into a pGEM®-T Easy (PROMEGA) vector. The fragments were cloned again in pBJ36-*NLS:3xGFP* (8) in front of the *NLS:3xGFP:NOSt:OCSt* cassette using *Pst*I and *Xma*I (NEB). pBJ36-*pChRCO::NLS:3xGFP* was modified by GenScript to generate pBJ36-*pChrco*^{enh}*::NLS:3xGFP*, harbouring the enhancer deletion present in the *rco*$^{enh-}$ mutant. All the expression cassettes were cloned in pMLBART using *Not*I. For *RCOenh::NLS:3xGFP, rcoenh*$^{Act69-}$*::NLS:3xGFP, rcoenh*$^{Rep64-}$*::NLS:3xGFP, hybrid_lmi1-rcoenh*^{mut14}*::NLS:3xGFP,* and *RCOenh*$^{*LMI1-like-dupl}$*::NLS:3xGFP,* we defined the enhancers as the sequence within -716 bp and -1347 bp from the *RCO* ATG for *RCOenh*^{500}, and the sequence within -1035 bp and -1550 bp from the *LMI1* ATG for *LMI1enh*^{500}. In *rcoenh*$^{Act69-}$*::NLS:3xGFP,* the *RCO* enhancer contains the deletion of Act69, as in *mut4*. In *rcoenh*$^{Rep64-}$*::NLS:3xGFP*, the *RCO* enhancer contains the deletion of Rep64, as in *mut5*. In *hybrid_lmi1-rcoenh*^{mut14}*::NLS:3xGFP,* the LMI1/RCO enhancer chimera mirrors the sequence recombination in *mut14*. In *RCOenh-LMI1-like-dupl::NLS:3xGFP*, the substituted region corresponds to the 27bp *LMI1* enhancer sequence between -1392 and -1419 from the *LMI1* ATG inserted replacing the 60bp *RCO* enhancer sequence between -1089 and -1149 bp from the *RCO*

ATG. All these sequences, were placed upstream of a minimal CAMV35S promoter (position -46 to +8 of the 35S promoter (9)), and were synthesized and cloned into pUC57 by GenScript. Then, the enhancer:m35S fragments were cut with *Pst*I and *Xma*I and ligated into pBJ36-NLS:3xGFP. The expression cassettes were subsequently digested with *Not*I and ligated into pMLBART.

Plant transformation and mutant selection

Plant transformation was based on the original floral dip protocol reported in Clough & Bent (10). 5 µl of Agrobacterium GV3101 culture transformed with a vector for binary transformation was used to inoculate 1L LB medium with appropriate antibiotics and was cultured at 28°C until an optical density of OD600nm=0.7-1 was reached. Cells were pelleted and resuspended in 800ml infiltration medium. 1 L medium was composed by: 50g sucrose (Sigma), 2.3g Murashige & Skoog Medium, including Gamborg's vitamins (Duchefa Biochem), and 0.05% Silwet L77. Inflorescences with unopened buds were submerged in the infiltration medium for 1 min and stored overnight in dark and contained conditions with opaque bags to preserve humidity. Plants were subsequently transferred to the greenhouse, and the resulting seeds (T0) were harvested once dried.

To select for BASTA® (Bayer Crop Science) resistant transgenic lines, T0 seeds were sown densely and grown on soil in the greenhouse. Plants were sprayed with 0.2% BASTA® solution when the cotyledons were expanded and the first leaf was visible (normally, 14 days after sowing). Plants were sprayed again between 3 days and 1 week after the first treatment. Transgene presence was validated through genotyping PCR on surviving T1 plants. For the CRISPR/Cas9 transformants, a first screen for mutations was performed in T1 plants. Homozygous mutations were identified in T2, T3, or T4 by checking the segregation ratio. Deletions or insertions were assessed by PCR and gel electrophoresis, and the presence of point mutations was evaluated by sequencing. Only individuals without the Cas9 transgene were kept.

Genotyping

Genomic DNA extraction

One young leaf or developing inflorescence from each plant was harvested in 1.5 ml Eppendorf tubes (Germany) or in Polypropylene 1.2mL Racked Collection Microtubes QIAvac (QIAGEN 19560) for DNA extraction. The leaf tissue was ground with a pestle or, for large scale, the tissue disruption was accomplished by shaking the 96-collection tubes, after adding metal beads to each tube, with a TissueLyser (QIAGEN) for 30 s at 300 rpm. After adding immediately 400 µl of 2X CTAB buffer, the samples were incubated at 65°C for 10 min, and 1 volume of chloroform/isoamylalcohol (24:1) was added. After vortexing for ca. 15 s, the samples were centrifuged for 5 min at 10000-15000xg (20 min, 5000-8000xg for Racked Collection Microtubes) to separate the aqueous phase from the organic one. 200 µl of the upper aqueous phase were collected and mixed with the same volume of isopropanol. After centrifuging at 10000-15000xg for 15 min (5000-8000xg, 1 h for Racked Collection Microtubes), the supernatant was discarded. The pellet was then washed once with 70% ethanol at 10000-15000xg centrifugation for 3 min (5000-8000xg, 15 min for Racked Collection Microtubes). The DNA pellet was air-dried overnight, dissolved in 100 µl of Milli-Q® (Merck) water for 10 min at 65°C, and stored at 4°C (short term) or -20°C.

Polymerase Chain Reaction (PCR)

PCRs were performed with the MangoTaq™ DNA Polymerase (Bioline) to amplify either genomic regions from extracted plant genomic DNA for genotyping or plasmid fragments from transformed *E. coli* or Agrobacterium strains for diagnostic colony PCR. The PCR program and reagent concentrations were calculated following the instructions of the manufacturer. Genotyping primers are reported in Supplemental Table S2.

SANGER sequencing

Sanger sequencing of purified PCR products was performed by the LIGHTRUN tube service offered by Eurofins Genomics (Germany). Sample preparation was performed following the company's instructions.

Table S2. Genotyping primers.

Primer name	Primer sequence	Use	Origin
A1-Ch_enh_LMI1_fw	TTCTTATGCACTGTCGTTAGTTTT	Amplification of *ChLMI1enh*^{500}, used to genotype the CRISPR mutants with *LMI1* enhancer mutation	This study
A2-Ch_enh_LMI1_rev	TGTGAACCAACCAATTCAAAG		This study
A3-Ch_enh_LMI1_fw_nest	TTTTAACATAGAAAAAGATTCCTCTCC		This study
A4-Ch_enh_LMI1_rev_nest	GGGGCATGTGCATATTTGAT		This study
A5-Ch_enh_RCO_fw	CCTTTACTCTCCATCGCTTACAA	Amplification of *ChRCOenh*^{500}, used to genotype the CRISPR mutants with *RCO* enhancer mutation	This study
A6-Ch_enh_RCO_rev	TGCTAGTTTGGCGCTATCAA		This study
A7-Ch_enh_RCO_fw_nest	TGCATCAAAATTACGCTGAG		This study
A8-Ch_enh_RCO_rev_nest	GGTTGCGTTCGCAAACTAAT		This study
A47-Cas9_fw	GTATGTATATATGTAGATCTGG	Genotyping transgene (Cas9) presence in CRISPR lines	This study
A48-Cas9_rv	GTATGTATATATGTAGATCTGG		This study
A86-upstr_LMI1_fw	ACCCCATAAAAAGCATCCAT	Genotyping of the *LMI1* enhancer mutations in *mut7* with A2 and of *mut15* with A6	This study
A84-far_upstr_LMI1_fw	TTTGGAATCGTTGTATTCTTCAA	Genotyping *lmi1*$^{-}$	This study
A93-LMI1_CDS_rv	TGAAGGGAAAAAGCTTGTCTTA		This study
A102-RCO_CDS_middle_fw	CCGCTTTCTCTTCTTCTTTTG	Genotyping *rco*$^{-}$; genotyping *pRCO::RCOg:Venus* in *rco*$^{enh-}$	This study
A103-RCO_CDS_rv	GGAACTCGTTTCATTGGATGT	Genotyping *rco*$^{-}$	This study
A106-LMI1-Venus_fw	CTTCTTGCTGGCAGCCTTAC	Genotyping *pLMI1::LMI1cds:Venus* in *lmi1*$^{enh-}$	This study
A107-LMI1-Venus_rv	AAGTCGTGCTGCTTCATGTG	Genotyping *pRCO::RCOg:Venus* in *rco*$^{enh-}$ and *pLMI1::LMI1cds:Venus* in *lmi1*$^{enh-}$	This study
A111-M13_fw	GTAAAACGACGGCCAGT	Genotyping of the reporter lines	This study
A112-M13_rv	GGAAACAGCTATGACCATG		This study
A196-zz133_ins_rv	CCATGGATCCAAGCTTATCG		This study

Quantitative RT PCR

Plants were grown for 14 days before the apical meristems with surrounding developing leaves (leaves from 1 to 4, already expanded at this stage, were removed) were dissected and collected in liquid nitrogen. 10 samples were pooled together to create one biological replica. RNA was

extracted using the RNeasy Plant Mini Kit (Qiagen 74904) following manufacturer's instructions. DNase I treatment was applied on-column during the RNA extraction by RNase-Free DNase Set (Qiagen 79254) following manufacturer's instructions. 400 ng of RNA was reverse-transcribed using the SuperScript® VILO™ cDNA Synthesis Kit (Invitrogen 11754050) following manufacture's instruction. qPCR was performed using Power SYBR Green Master Mix (Thermo Scientific 4367659) in QuantStudio 5 (384 wells) or QuantStudio 3 (96 wells) (Thermo Scientific). Three or four biological replicates were performed in triplicate (three technical replicates for each biological replicate). The expression of GAPDH was used to normalize the expression of CT values. Relative expression levels were determined by the comparative CT (ΔΔCT) method (11). Primers used in qRT-PCR experiments, reported in Table S3, are as in Vlad et al. (12).

Table S3 Primers used for RT-qPCR.

**Primer name**	**Primer sequence**	**Use**	**Origin**
A15-Ch_GAPDH_qPCR_fw	TGACCACCGTCCACTCCATCAC	qRT *ChGAPDH* housekeeping gene primers	Vlad et al., 2014
A16-Ch_GAPDH_qPCR_rv	GCTCTTCCACCTCTCCAGTCCTTC		Vlad et al., 2014
A27-Ch_LMI1_qPCR_fw	GTGCATCCTGACATTCTTAGCACT	qRT *ChLMI1* primers	Vlad et al., 2014
A28-Ch_LMI1_qPCR_rv	CGCAATCAGCATCCCAAATAT		Vlad et al., 2014
A29-Ch_RCO_ex2_qPCR_fw	GCCACCTGGGTTTAGGTTTC	qRT *ChRCO* primers	Vlad et al., 2014
A30-Ch_RCO_ex2_qPCR_rv	GCACAGGAGAAATTGGAGTCA		Vlad et al., 2014

Scanning Electron Micrograph imaging

Freshly dissected live tissues were mounted directly on conductive carbon adhesive tabs and imaged. Scanning electron microscopy was performed on a Hitachi FlexSEM 1000 operated in variable-pressure (VP-SEM) mode to limit charging of uncoated hydrated samples. Short dwell times were used to preserve surface detail.

Sample preparation and confocal microscopy imaging

Seeds of *A. thaliana* and *C. hirsuta* T2 transgenic lines were sowed on soil and grown for 10-13 days in LD conditions. When leaf 6 size was between 300-500 µm, seedlings for confocal imaging were dissected with tweezers and needles under a stereoscope to expose leaf 6 and mounted in

water without fixation. Confocal imaging was performed with an SP8 upright confocal laser microscope (Leica) equipped with a long working-distance water immersion objective (AP 25x/0.95). All images were taken with a pinhole of 1 AU. The chlorophyll autofluorescence detection window used was in the 650-750 nm range. Additional imaging settings are reported in Table S4.

Image registration and analysis

Registration of transformed GFP expression patterns

10 samples from independent T2 lines in both *C. hirsuta* Oxford and *A. thaliana* Col-0 of *RCOenh::NLS:3xGFP, rcoenh^{Act64-}::NLS:3xGFP* (9 *C. hirsuta* samples for this construct), *rcoenh^{Rep69-}::NLS:3xGFP,* and *hybrid_lmi1-rcoenh^{mut14}::NLS:3xGFP* (8 *C. hirsuta* samples for this construct) were imaged with strictly identical settings as indicated in Table S4.

Table S4. Confocal parameters for GFP expression imaging registration.

**Paramenters**	**Settings**
Solid-state laser	2.5% output, 488 nm excitation wavelength
HyB	496-523 nm for GFP detection,<br>25% smart gain
Scanner speed	600 Hz, bidirectional
Line averaging	2
Image averaging	1
Consequential scan	No
Zoom	No
Z-step size	1 µm
Image size	1024x1024
Bit per pixel	8-bit

All images were processed in Fiji (13), except for the creation of reference shapes and a final mask outside of these reference shapes, which were performed in Photoshop (Adobe). The maximum projection of the GFP signal was mapped onto reference shapes of representative *Arabidopsis thaliana* and *Cardamine hirsuta* developing leaves using the “Transform > Landmark Correspondences” plugin. 10 landmarks were used for *A. thaliana* and 24 for *C. hirsuta*, placed at clearly identifiable features of the developing leaves (see Tables S5 and S6, respectively). The

plugin settings for the transformation are detailed in Table S7. The transformed images were then imported as an image sequence, and an average-intensity projection was generated. To enhance the readability of the resulting patterns while maintaining comparability among samples, the following additional steps were applied to the average-intensity outputs for the purpose of visualization: 1. To make low-expression profiles (e.g., *rcoenh*$^{Act64-}$*::NLS:3xGFP*) more visible, pixel values were resampled by rescaling the maximum intensity to 100. 2. A Gaussian blur was applied to reduce excessively bright nuclear signals from individual samples that were still detectable in the averaged projection. Without this step, these residual signals appeared as granular, cell-like artifacts that could be mistaken for real subcellular signals. This step was executed using *Process* > *Filters* > *Gaussian Blur…* with a Sigma (Radius) value of 10 pixels. Finally, the "fire" look-up table was applied.

Table S5. *A. thaliana* landmarks.

Landmark	Position
1	On the abaxial side at the base of the primordium, ideally placed centrally in relation to the medio-lateral axis of the leaf.
2	Centrally aligned with the medio-lateral axis, placed on the abaxial side at the same position along the proximo-distal axis as the upper sinus of the first serration.
3	Tip of the primordium.
4	Halfway along the margin, between the tip of the primordium and the upper sinus of the first serration.
5	Upper sinus of the first serration.
6	Tip of the first serration.
7	Lower sinus of the first serration.
8	Tip of the second serration.
9	Lower sinus of the second serration.
10	At the base of the primordium along the margin, ideally aligned with landmark 1 along the medio-lateral axis.

Table S6. *C. hirsuta* landmarks.

Landmark	Position
1	On the abaxial side at the base of the primordium, ideally placed centrally in relation to the medio-lateral axis.
2	On the abaxial side, aligned with the lower sinus of the second lateral leaflet along the proximo-distal axis. Centrally placed in relation to the medio-lateral axis.
3	On the abaxial side, aligned with the lower sinus of the first lateral leaflet along the proximo-distal axis. Centrally placed in relation to the medio-lateral axis.

Landmark	Position
4	On the abaxial side, aligned with the upper sinus of the first lateral leaflet along the proximo-distal axis. Centrally placed in relation to the medio-lateral axis.
5	On the abaxial side, at the base of the developing terminal leaflet, ideally placed at the lowest point of the concave depression characteristic of the abaxial side in this area. Centrally placed in relation to the medio-lateral axis.
6	On the abaxial side, halfway between the previous landmark and the tip of the developing primordium, centrally placed in relation to the medio-lateral axis.
7	Tip of the developing primordium.
8	Lowest point of the concave curvature of the terminal serration of the developing terminal leaflet, along the margin.
9	Sinus beneath the apical serration of the terminal leaflet.
10	Tip of the serration sometimes present between the two main serrations of the developing leaflet. If not present, place it near the previous landmark in the sinus.
11	Sinus beneath the serration sometimes present between the two main serrations of the developing leaflet. If not present, place it near the previous landmark in the sinus.
12	Tip of the second main serration of the developing leaflet.
13	Second maxima (where the slope of the margin changes convexly) of the second main serration.
14	Slight depression (where the slope of the margin changes concavely) occurring when a secondary serration emerges as the last one from the top along the margin.
15	Lower tip, sometimes a proper serration, or simply a point where the slope of the margin line changes drastically (convex) towards the upper sinus of the first lateral leaflet.
16	Point where the slope of the margin line changes.
17	Upper sinus of the first lateral leaflet.
18	Upper lateral tip of the first lateral leaflet.
19	Tip of the first lateral leaflet.
20	Lower lateral tip of the first lateral leaflet.
21	Lower sinus of the first lateral leaflet.
22	Tip of the second lateral leaflet.
23	Lower sinus of the second lateral leaflet.
24	Base of the leaf along the margin, ideally aligned with landmark 1 along the medio-lateral axis.
25 (optional)	Margin, halfway between landmarks 16 and 17.
26 (optional)	Margin, halfway between landmarks 17 and 18.

Table S7. Settings for the transformation of GFP maximum projection signals with the Landmark Correspondences Fiji plugin.

Paramenters	Settings
Transformation method	Moving Least Squares (non-linear)

Alpha	1
Mesh resolution	32
Transformation class	Affine

Quantification Along the Leaf Margin

To quantify the expression patterns specifically at the leaf margin, a segmented line region of interest (ROI) was appropriately designed on the *A. thaliana* and *C. hirsuta* developing leaf reference shapes (see Fig. S8). This ROI was then applied to each individual transformed maximum projections, and pixel intensities along the ROI were measured using the Plot Profile function in Fiji.

***In situ* hybridization assays**

RNA *in situ* hybridizations were performed on 8 µm longitudinal sections of fixed and paraffin-embedded young *C. hirsuta* Ox and CRISPR/Cas9-generated mutant shoot apices with primordial leaves largely as previously described (12). For these experiments, plants were grown in short-day conditions (8h light, 16h dark cycle) and for 26-29 days post germination. Digoxigenin-labelled (DIG RNA Labeling Mix, Roche) antisense RNA probes to *C. hirsuta LMI1* and *RCO* coding sequences were generated using cDNA templates obtained after amplification with the primer combinations 5’-AAATCCATACACACATGCTGATATG-3’, 5’-GTGCgtaatacgactcactatagggcGGTGGTTAGATTCTCTGTTCTTGG-3’ and 5’-AATTCGTTTACGCCTGCCGC-3’, 5’-GTGCgtaatacgactcactatagggcTAAGGAAAAGCCTGAGATATCGC-3’, respectively (T7 RNA polymerase binding motif in the reverse primers indicated in lowercase). After hybridization and washing, the sections were covered with 70% 2,2-thiodiethanol (TDE) at pH = 9 and imaged with a Zeiss Axio Imager equipped with a digital color camera and Differential Interference Contrast (DIC) optics. To cover a broader hybridization pattern, images of three consecutive sections were registered and minimum projections were generated using the image processing package Fiji (13).

Leaf phenotype analysis

Leaf shape acquisition

Leaves from 5- to 6-week-old plants grown in soil in LD were adhered to paper using clear self-adhesive polyester foils (VMR, 731-0311) and were digitally scanned at 800 or 600 dpi 24-bit color using a PERFECTION v700 (EPSON) photo scanner. Leaf silhouettes were created in Photoshop with careful supervision and manual corrections, in order to precisely match the leaf shape. Final shapes for analysis (black on white background) were exported at 600 dpi.

Leaf quantification

Measurements were performed in Fiji. Formulas used to calculate the quantifiers shown in this study are indicated in Table S8.

Table S8. Calculation of the leaf shape quantifiers used in this study

Quantifier	Formula	Reference
Stipule Outgrowth Index	$1-\frac{length\ leaf - length\ total\ stipules}{length\ leaf + length\ total\ stipules}$; Average of the values of all the primary leaves	This study
NDMC	$\frac{leaf\ perimeter - convex\ hull\ perimeter}{leaf\ perimeter + convex\ hull\ perimeter}$	Leigh et al.(14)
Ratio min/max width leaflet	$\frac{\max width\ leaflet - \min width\ leaflet\ (petiolule)}{\max width\ leaflet + \min width\ leaflet\ (petiolule)}$	This study

Bioinformatics, quantification and statistical analysis

Enhancer alignment

The alignment has been performed on 14 *RCO* and 17 *LMI1* enhancer sequences within the Brassicaceae family (species and NCBI assembles used are indicated in Table S9) with BAli-phy (15) version 3.6.1. Run settings: 100,000 iterations with burning of the first 1000. Substitution model: TN. The full alignment is reported in Dataset S5.

Table S9. Sequences used in the alignment.

Species	Enhancer	BioSample	BioProject
Euclidium syriacum	*RCO*	SAMEA4527703	PRJEB16743
Aurinia saxatilis	*RCO*	SAMEA4640837	PRJEB26555

Pseudoturritis turrita	*RCO*	SAMEA4643371	PRJEB26555
Arabis alpina	*RCO*	SAMEA4527704	PRJEB16743
Arabis montbretiana	*RCO*	SAMN02983095	PRJNA258048
Kernera saxatilis	*RCO*	SAMEA4640850	PRJEB26555
Sisymbrium irio	*RCO*	SAMN02152446	PRJNA202979
Eutrema salsugineum	*RCO*	SAMN02981485	PRJNA73205
Cardamine hirsuta	*RCO*		
Leavenworthia alabamica	*RCO*	SAMN02146679	PRJNA202983
Capsella rubella	*RCO*	SAMN02981483	PRJNA13878
Turritis glabra	*RCO*	SAMEA4643370	PRJEB26555
Arabidopis lyrata	*RCO*	SAMN02981250	PRJNA41137
Arabidopsis halleri	*RCO*	SAMEA3885198	PRJEB12914
Aethionema arabicum	*LMI1*	SAMN02169162	PRJNA202984
Euclidium syriacum	*LMI1*	SAMEA4527703	PRJEB16743
Alyssum argenteum	*LMI1*	SAMEA4639485	PRJEB26555
Aurinia saxatilis	*LMI1*	SAMEA4640837	PRJEB26555
Pseudoturritis turrita	*LMI1*	SAMEA4643371	PRJEB26555
Arabis alpina	*LMI1*	SAMEA4527704	PRJEB16743
Arabis montbretiana	*LMI1*	SAMN02983095	PRJNA258048
Kernera saxatilis	*LMI1*	SAMEA4640850	PRJEB26555
Sisymbrium irio	*LMI1*	SAMN02152446	PRJNA202979
Eutrema salsugineum	*LMI1*	SAMN02981485	PRJNA73205
Cardamine hirsuta	*LMI1*		
Leavenworthia alabamica	*LMI1*	SAMN02146679	PRJNA202983
Capsella rubella	*LMI1*	SAMN02981483	PRJNA13878
Turritis glabra	*LMI1*	SAMEA4643370	PRJEB26555
Arabidopis lyrata	*LMI1*	SAMN02981250	PRJNA41137
Arabidopsis halleri	*LMI1*	SAMEA3885198	PRJEB12914
Arabidopsis thaliana	*LMI1*	SAMN03081427	PRJNA10719

Sliding window alignment (Fig. S9A)

Sequence conservation to the *C. hirsuta RCO* enhancer was computed on the enhancer alignment using 10-bp windows (1-bp step) along ungapped reference coordinates; query gaps were treated

as mismatches (16). Analyses and plotting were carried out in R with the Biostrings and ggplot2 packages.

Binding site prediction

Transcription factor binding site predictions were performed using the default settings of the Binding Site Prediction tool from the PlantRegMap web server (17), applying its internal *A. thaliana* transcription factor binding motif database. The genomic sequences corresponding to the Rep64 and Act69 regions were used as input. Predictions were filtered using a p-value cutoff of 1e-4.

Statistical Analysis

All statistical analyses were performed in R using base R or the package *afex*. When datasets included uneven group sizes or multiple experiments (batches), linear mixed-effects models were fitted with *lmer*. In these models, genotype was treated as a fixed effect and batch as a random effect, where the latter refers to data collected from independent experiments under comparable growth conditions. For simple balanced datasets standard linear models were fitted using *lm* provided by base R. Normality and homogeneity of residuals were assessed by inspecting Q–Q plots, histograms, and residuals vs. fitted plots to verify if the assumptions of linear modeling were met, and to asses goodness of fit.

Normalized leaf area for node effects (Fig. 3H)

Fig. 3H shows leaf areas of nodes 5, 6, 7, and 8 adjusted for the effect of leaf node. To calculate this, we first fitted the following linear mixed effects model using *lmer* to test for significant genotype effects:

Area ~ genotype + leaf node + (1 | batch)

We then fitted the model without the genotype term to calculate corrected leaf areas as the sum of the intercept and the residuals of this model. The corrected area data was then plotted in Fig. 3H.

Table S10. Materials and Resources used in the study.

REAGENT or RESOURCE	SOURCE	IDENTIFIER
Bacterial and virus strains		
Agrobacterium radiobacter strain GV3101(pMP90)	MPIPZ stock	N/A
Escherichia coli DH10B	MPIPZ stock	N/A
Chemicals, peptides, and recombinant proteins		
1/2 MS Medium including vitamins	Duchefa Biochem	Cat# M0222.0050
BASTA®	Bayer Crop Science	Cat# 06470033
Silwet L77	Obermeier GmbH & Co.	Cat# #7060-10
Critical commercial assays		
Mango Taq™ DNA Polymerase	Bioline	Cat# BIO-21083
DIG RNA labelling mixture	Roche/Sigma-Aldrich	Cat# 11277073910
RNeasy Plant Mini Kit	QIAGEN	Cat# 74904
SuperScript® VILO™ cDNA Synthesis Kit	Invitrogen	Cat# 11754-050
Power SYBR™ Green PCR Master Mix	Thermo Fisher	Cat# 4367659
Gateway™ LR Clonase™ II Enzyme mix	Thermo Fisher	Cat# 11791020
Experimental models: Organisms/strains		
A. thaliana: Columbia ecotype (Col-0)	MPIPZ stock	N/A
A. thaliana: *pChLMI1::NLS:3xGFP*	This study	N/A
A. thaliana: *pChRCO::NLS:3xGFP*	This study	N/A
A. thaliana: *RCOenh::NLS:3xGFP*	This study	N/A
A. thaliana: $rcoenh^{Act69-}$*::NLS:3xGFP*	This study	N/A
A. thaliana: $rcoenh^{Rep64-}$*::NLS:3xGFP*	This study	N/A
A. thaliana: hybrid_lmi1-$rcoenh^{mut14}$*::NLS:3xGFP*	This study	N/A
C. hirsuta: Oxford ecotype (Ox)	Hay and Tsiantis (1)	Miltos Tsiantis, MPIPZ
C. hirsuta: $lmi1^{-}$	This study	N/A
C. hirsuta: $lmi1^{-}$ × *pChRCO::NLS:3xGFP*	This study	N/A
C. hirsuta: *lmi1*; rco^{mut14}	This study	N/A
C. hirsuta: *lmi1*; rco^{mut15}	This study	N/A
C. hirsuta: $lmi1^{enh-}$	This study	N/A
C. hirsuta: $lmi1^{enh-}$/+	This study	N/A
C. hirsuta: $lmi1^{enh-}$; *pChLMI1::ChLMI1cds:Venus*	This study	N/A
C. hirsuta: $lmi1^{mut5}$; rco^{mut5}	This study	N/A
C. hirsuta: $lmi1^{mut5}$/+; rco^{mut5}/+	This study	N/A
C. hirsuta: $lmi1^{mut5}$/-; rco^{mut5}/-	This study	N/A
C. hirsuta: $lmi1^{mut6}$; rco^{mut6}	This study	N/A
C. hirsuta: $lmi1^{mut6}$/+; rco^{mut6}/+	This study	N/A
C. hirsuta: $lmi1^{mut6}$/-; rco^{mut6}/-	This study	N/A
C. hirsuta: $lmi1^{mut7}$; rco^{mut7}	This study	N/A
C. hirsuta: $lmi1^{mut7}$/+; rco^{mut7}/+	This study	N/A
C. hirsuta: $lmi1^{mut7}$/-; rco^{mut7}/-	This study	N/A
C. hirsuta: $lmi1^{mut8}$	This study	N/A
C. hirsuta: $lmi1^{mut9}$	This study	N/A
C. hirsuta: $lmi1^{mut10}$	This study	N/A

C. hirsuta: $lmi1^{mut11}$	This study	N/A
C. hirsuta: $lmi1^{mut12}$	This study	N/A
C. hirsuta: $lmi1^{mut12}$	This study	N/A
C. hirsuta: $lmi1^{mut12}$	This study	N/A
C. hirsuta: *rco*	Vlad et al. (12)	Miltos Tsiantis, MPIPZ
C. hirsuta: rco^{enh-}	This study	N/A
C. hirsuta: $rco^{enh-}/+$	This study	N/A
C. hirsuta: $rco^{enh-}/-$	This study	N/A
C. hirsuta: rco^{enh-}; *pChRCO::ChRCO:Venus*	This study	N/A
C. hirsuta: rco^{mut1}	This study	N/A
C. hirsuta: rco^{mut2}	This study	N/A
C. hirsuta: $rco^{mut2}/+$	This study	N/A
C. hirsuta: $rco^{mut2}/-$	This study	N/A
C. hirsuta: rco^{mut3}	This study	N/A
C. hirsuta: $rco^{mut3}/+$	This study	N/A
C. hirsuta: $rco^{mut3}/-$	This study	N/A
C. hirsuta: rco^{mut4}	This study	N/A
C. hirsuta: $rco^{mut4}/+$	This study	N/A
C. hirsuta: $rco^{mut4}/-$	This study	N/A
C. hirsuta: *pChLMI1::NLS:3xGFP*	This study	N/A
C. hirsuta: *pChRCO::NLS:3xGFP*	This study	N/A
C. hirsuta: $pChrco^{enh-}$*::NLS:3xGFP*	This study	N/A
C. hirsuta: *RCOenh::NLS:3xGFP*	This study	N/A
C. hirsuta: $rcoenh^{Act69-}$*::NLS:3xGFP*	This study	N/A
C. hirsuta: $rcoenh^{Rep64-}$*::NLS:3xGFP*	This study	N/A
C. hirsuta: $hybrid\_lmi1\text{-}rcoenh^{mut14}$*::NLS:3xGFP*	This study	N/A
C. hirsuta: $RCOenh*^{LMI1\text{-}like\text{-}dupl}$*::NLS:3xGFP*	This study	N/A
Oligonucleotides		
All oligos	N.A.	Tables S3 and S4
Recombinant DNA		
Vector: pDE-Cas9	Fauser et al.(3)	Addgene plasmid #61433
Vector: pMLBart	Gleave (18)	Miltos Tsiantis, MPIPZ
Vector: pBJ36-*NLS:3xGFP*	Zhang et al.(8)	N/A
Plasmid: pDE-Cas9_L4-L5	This study	N.A.
Plasmid: pDE-Cas9_L3-L6	This study	N.A.
Plasmid: pDE-Cas9_R3-R4-R5-R6	This study	N.A.
Plasmid: pDE-Cas9_R1-R2-R7-R8	This study	N.A.
Plasmid: pDE-Cas9_L3-L4-L5-L6	This study	N.A.
Plasmid: pDE-Cas9_L1-L2-L7-L8	This study	N.A.
Plasmid: pDE-Cas9_L3-L4-L5-L6-R3-R4-R5-R6	This study	N.A.
Plasmid: pDE-Cas9_LN1-LN2-LN3-LN4	This study	N.A.
Plasmid: *pChLMI1::ChLMI1cds:Venus*	This study	N.A.
Plasmid: *pChRCO::ChRCOg:Venus*	This study	N.A.

Plasmid: *pChLMI1::NLS:3xGFP*	This study	N.A.
Plasmid: *pChRCO::NLS:3xGFP*	This study	N.A.
Plasmid: *pChrco*$^{enh-}$*::NLS:3xGFP*	This study	N.A.
Plasmid: *RCOenh::NLS:3xGFP*	This study	N.A.
Plasmid: *rcoenh*$^{Act64-}$::NLS:3xGFP	This study	N.A.
Plasmid: *rcoenh*$^{Rep69-}$*::NLS:3xGFP*	This study	N.A.
Plasmid: *hybrid_lmi1-rcoenh*^{mut14}*::NLS:3xGFP*	This study	N.A.
Plasmid: *RCOenh**$^{LMI1\text{-}like\text{-}dupl}$*::NLS:3xGFP*	This study	N.A.
Software and algorithms		
BAli-Phy 3.6.1	Redelings (15)	https://www.bali-phy.org/
Fiji (Fiji is just ImageJ) (version 2.13.0 and following releases)	Schindelin et al. (13)	https://imagej.net/software/fiji/
Leica application suite X (LAS X)	Leica	https://www.leica-microsystems.com/products/ microscope-software/p/leica-las-x-ls/; RRID: SCR_013673
Photoshop (version CC 2019 and following releases)	Adobe	adobe.com
R (version 3.6.0 and following releases)	R Core Team	www.r-project.org; RRID: SCR_001905
R package: afex (version 1.4-1)	Singmann et al. (19)	https://CRAN.R-project.org/package=afex
R package: Biostrings (Bioconductor version 3.21)	Pagès et al. (20)	https://bioconductor.org/packages/release/bioc/html/Biostrings.html
R package: ggplot2 (version 3.3.5)	Wickham (21)	https://ggplot2.tidyverse.org; RRID: SCR_021139
PlantRegMap	Tian et al. (22)	https://plantregmap.gao-lab.org
Other		
C. hirsuta genome v1.0	Gan et al. (23)	https://gbrowse.mpipz.mpg.de/cgi-bin/gbrowse/chi1_public/

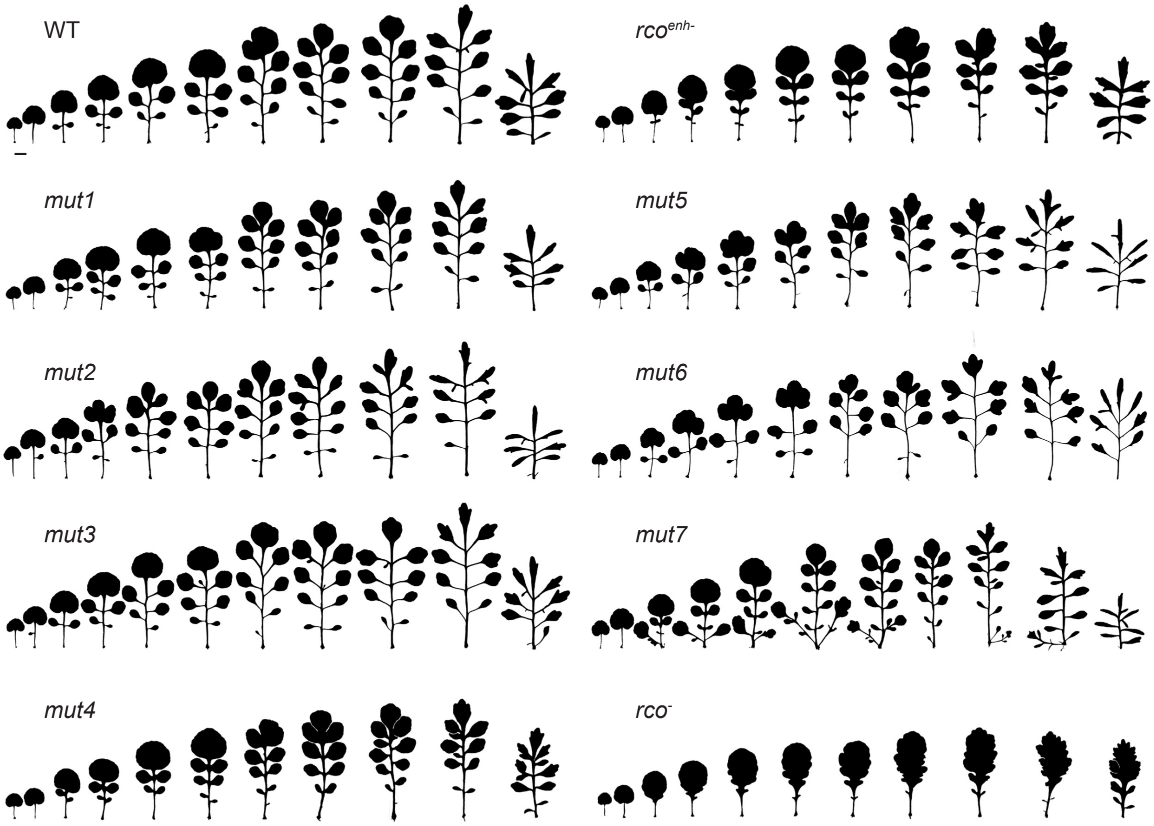

Fig. S1. Heterochronic leaf series of the *RCO^{enh}* alleles. Representative leaf series depicting leaf 1 to leaf 11, for wt, *mut1*, *mut2*, *mut3*, *mut4*, *rco^{enh-}*, *mut5*, *mut6*, *mut7* and *rco^{-}*. Scale bar: 1 cm.

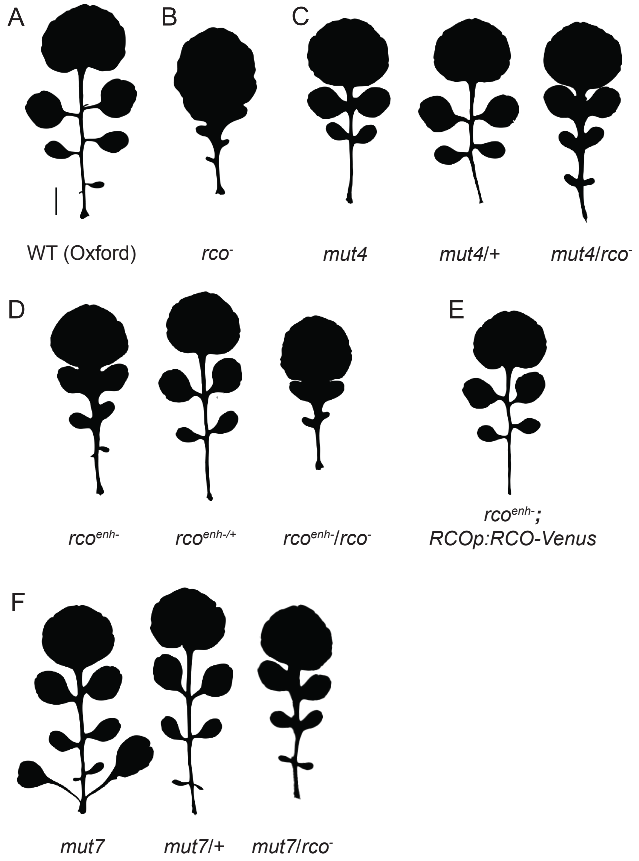

Fig. S2. Allelism tests and transgenic complementation for the *RCO*^{enh} loss-of-function alleles. Representative leaf 5 of **(A)** WT, **(B)** *rco*$^{-}$, **(C)** mut4, mut4/+, mut4/ *rco*$^{-}$, **(D)** *rco*$^{enh-}$, *rco*$^{enh-}$/+, *rco*$^{enh-}$/ *rco*$^{-}$, **(E)** *rco*$^{enh-}$*/-; RCOp:RCO-Venus,* **(F)** mut7, mut7/+, mut7/ *rco*$^{-}$, Note that the *RCO* loss-of-function phenotype becomes more severe when the CRISPR alleles exhibiting different degrees of mild loss-of-function phenotypes are crossed with the reference *RCO* loss-of-function allele *rco*$^{-}$.Scale bar: 1 cm.

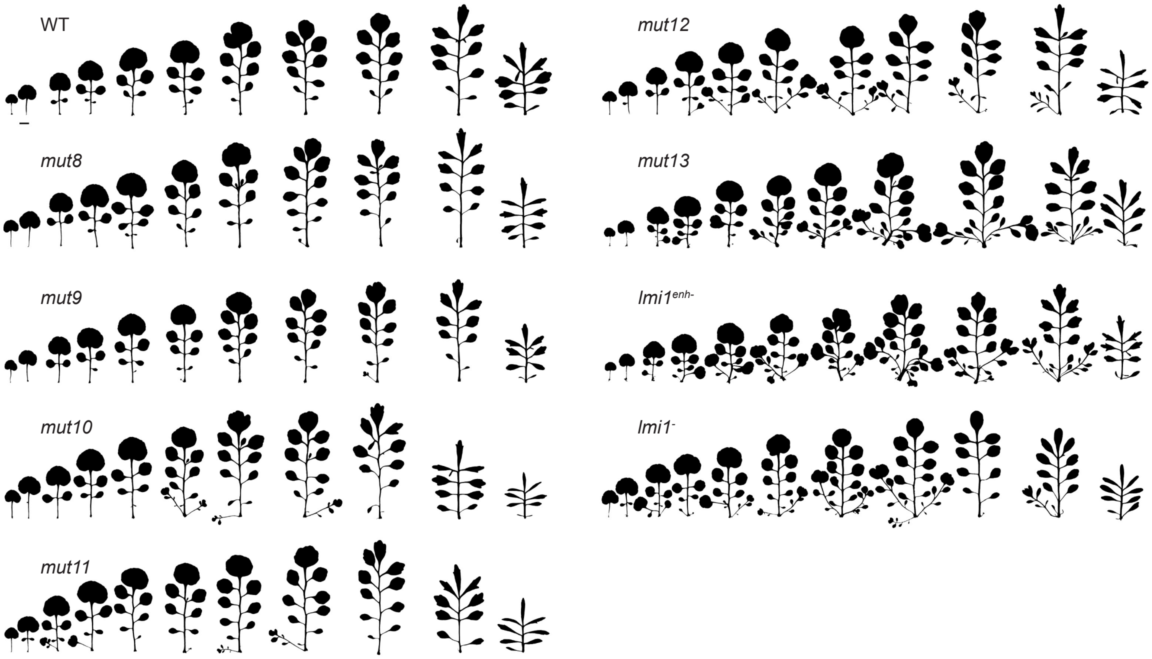

Fig. S3. Heterochronic leaf series of the *LMI1^{enh}* alleles. Representative leaf series depicting leaf 1 to leaf 11, for wt, *mut8*, *mut9*, *mut10*, *mut11*, *mut12*, *mut13*, *lmi1^{enh-}* and *lmi1^{-}*. Note the stipules grown as leaves along the main leaf's rachis. Scale bar: 1 cm.

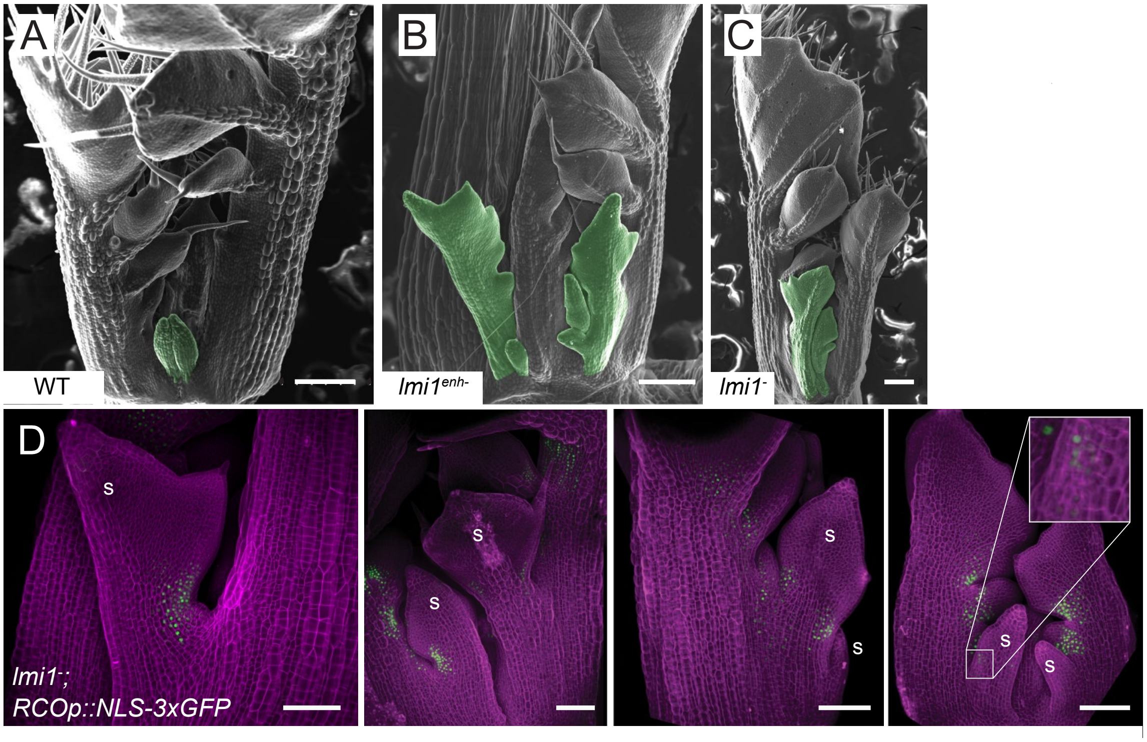

Fig. S4. LMI1 loss-of-function in *C. hirsuta* leaves is characterized by stipules mis-developing into leaves. (A), **(B)**, and **(C)** Scanning Electron Micrographs of developing leaves and stipules (pseudo-colored green) of WT, *lmi1*$^{enh-}$, and *lmi1*$^{-}$, respectively. **(D)** Expression of *RCOp::NLS-3xGFP* in *lmi1*$^{-}$ in developing leaves and stipules ("s" for stipules). The transition toward leaf development results in *RCO* expression (green signal) in the outgrowing stipules. Magenta: Propidium Iodide (PI) staining. Scale bars in (A)-(C): 200 μm. Scale bars in (D): 100 μm.

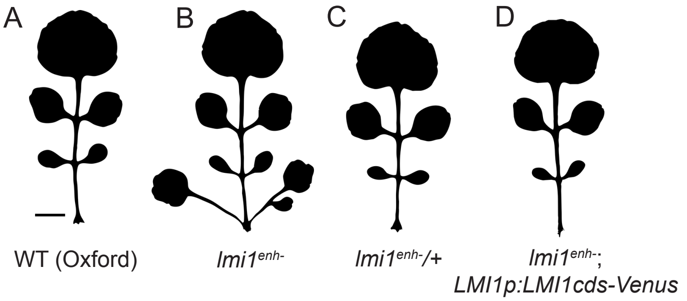

Fig. S5. The *LMI1* loss-of-function phenotype (stipules growing into leaves) caused by the deletion of the *LMI1* enhancer in *lmi1*$^{enh-}$ behaves as a recessive trait and can be complemented by expressing *LMI1* in the LMI1 domain. (A), **(B)**, **(C)**, and **(D)** representative leaf 5 of WT, *lmi1*$^{enh-}$, *lmi1*$^{enh-}$/+, and *lmi1*$^{enh-}$; *LMI1p:LMI1cds-Venus* plants.

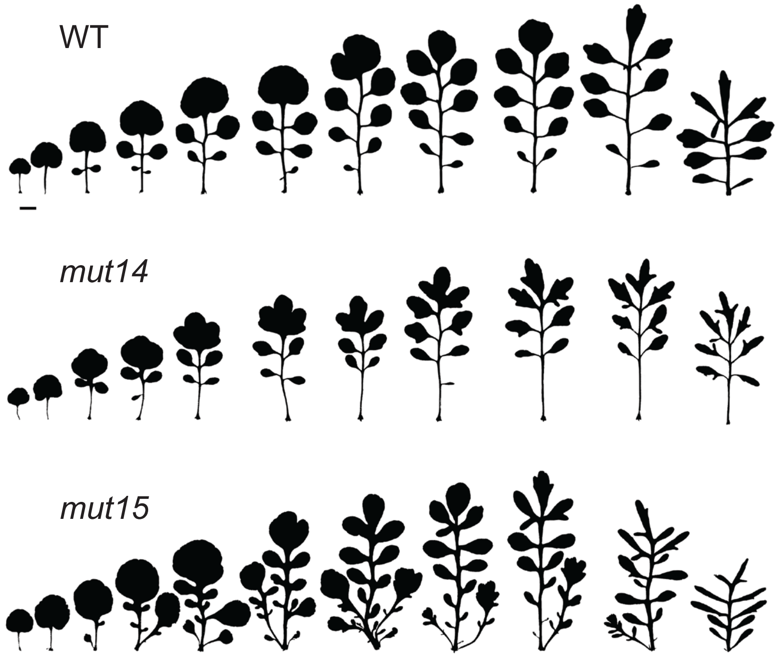

Fig. S6. Heterochronic leaf series of *LMI1/RCO* enhancer fusion alleles. Representative leaf series depicting leaf 1 to leaf 11, for wt, *mut14* and *mut15*. Scale bar: 1cm

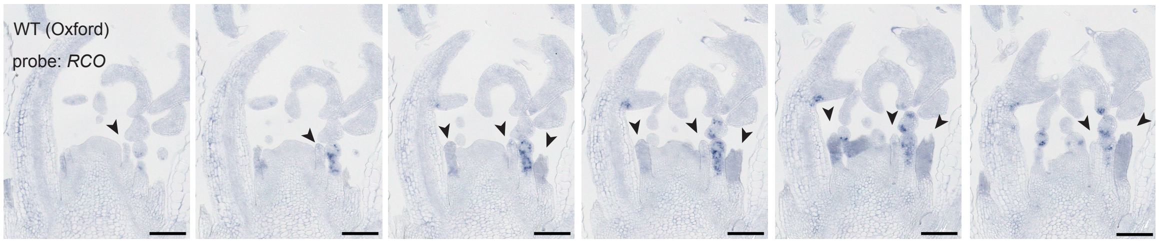

Fig. S7. *RCO* is not expressed in the stipules of WT *C. hirsuta* plants. Micrographs from *in situ* hybridization targeting the *RCO* transcript in consecutive vegetative meristem sections of a WT *C. hirsuta* (Ox) meristem. *RCO* is not detected in the stipules. Black arrowheads: stipules. Scale bar: 100 μm.

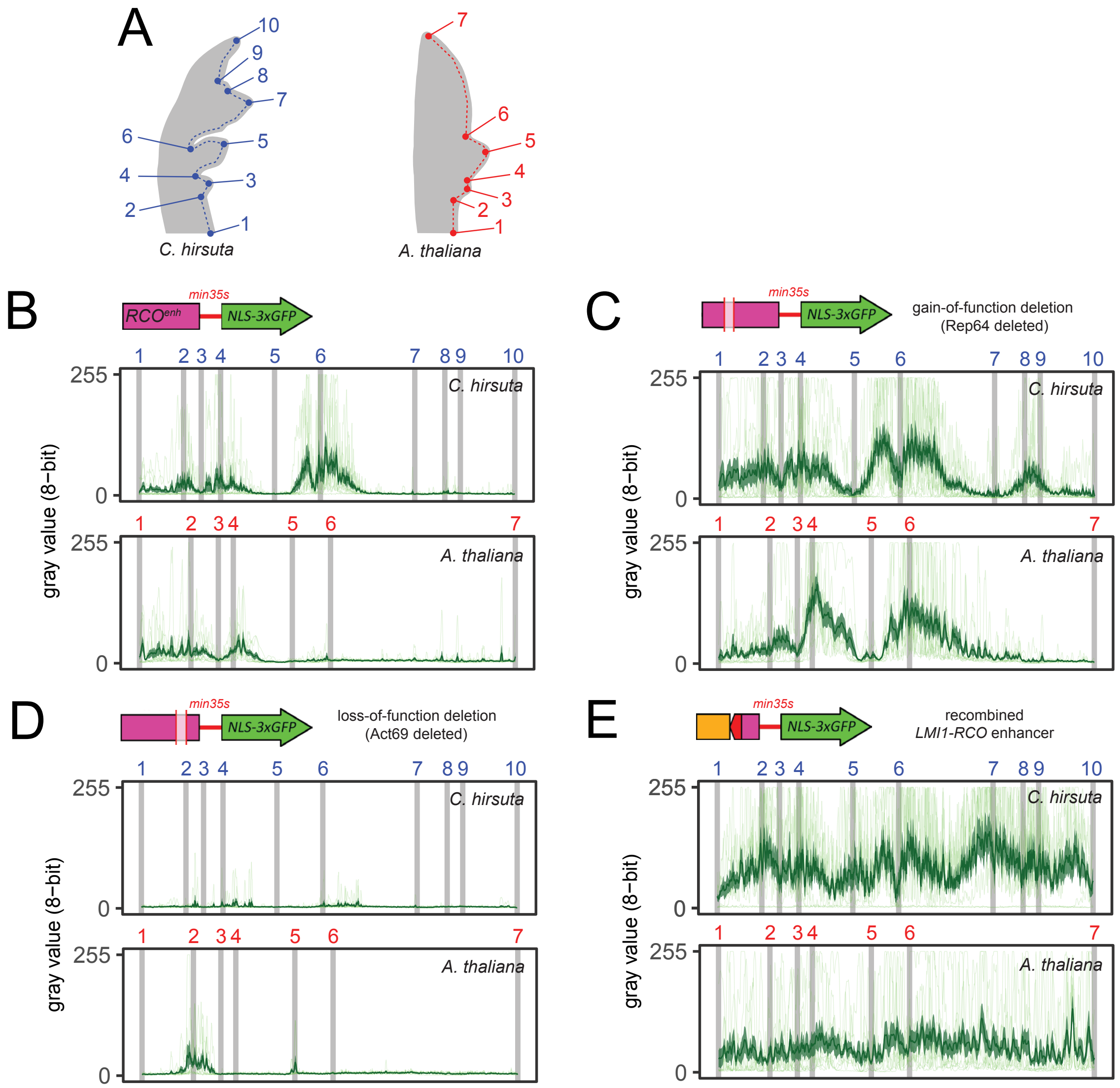

Fig. S8. Quantification of signal intensity in transgenic reporter lines recapitulating the deletions of region Rep64, Act69, and the hybrid enhancer in *mut14*. (A) Schematic showing the margin of the reference primordia for *C. hirsuta* (left) and *A. thaliana* (right) used to register the average signals shown in Fig. 4 B-E. **(B)**, **(C)**, **(D)**, **(E)** the pixel values (8-bit) of the registered maximum projections used to produce the average shown in Fig. 4 panels C'-F'. Light green: values of the independent samples. In darker green shades are reported also the +- SD (ribbon) and the average (thicker line). The position of landmarks shown in panel A is indicated. Landmarks were chosen as follows. *C. hirsuta* (left, blue): 1. Base, 2. Third sinus, 3. Second lateral leaflet's tip, 4. Second sinus, 5. First lateral leaflet's tip, 6. First sinus, 7. Lateral tip on terminal leaflet, 8. and 9. sinus(es) in the terminal leaflet, 10. Apex. *A. thaliana* (right, red): 1. Base, 2. Third sinus, 3. Second serration's tip, 4. Second sinus, 5. First serration's tip, 6. First sinus, 7. Apex.

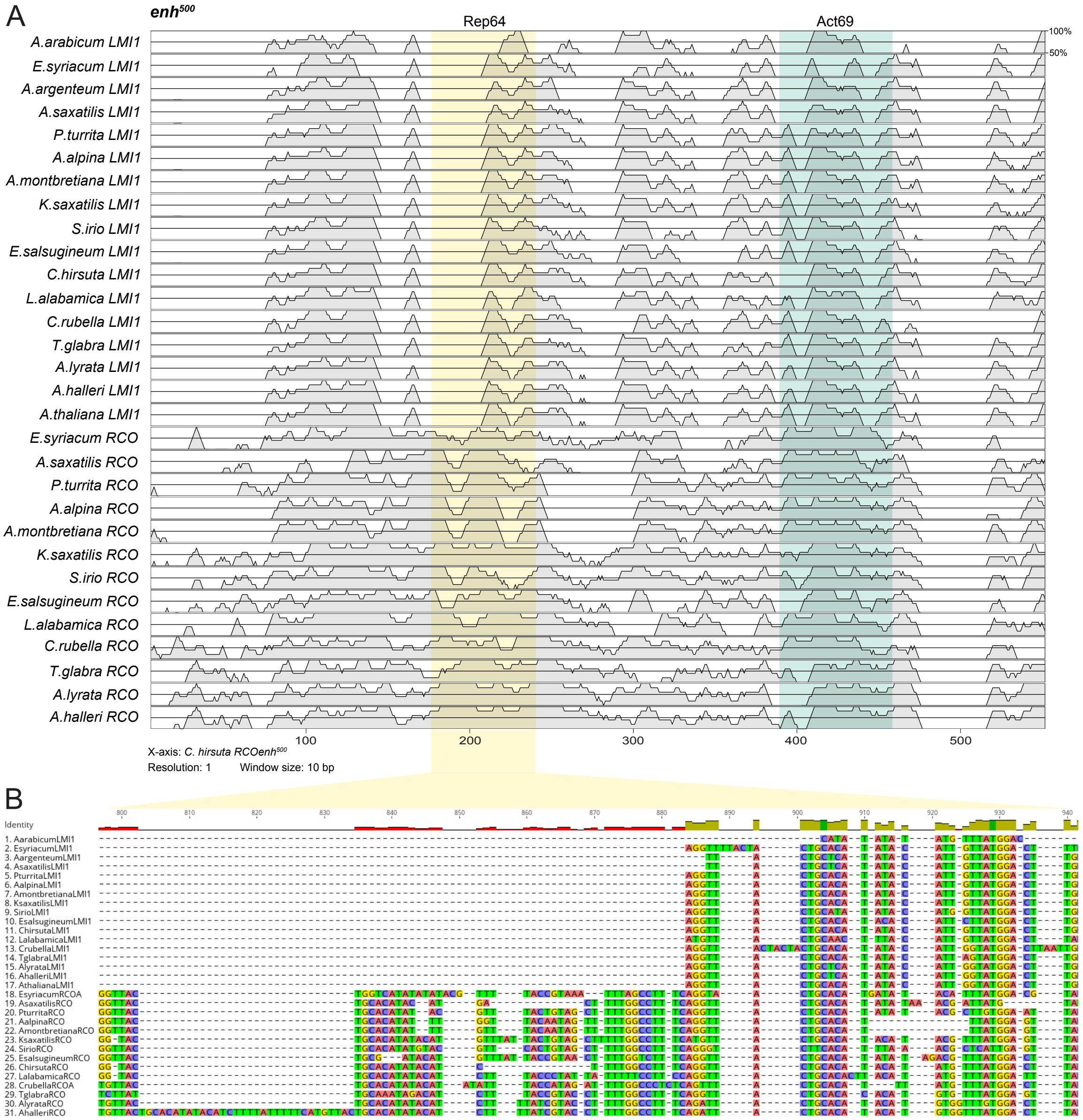

C *C. hirsuta RCOenh*^{500}

GAAATATGTAACAATTTGAATTCTTAATATTTACAAGTCCAGTTTGCTACTAAATTTTATTTTTAAAATTTTTTTGGTTATAATTTGATTTCATC
ACTAAAAGTTAAATTTAAATTCTTCAATTTTACTTTTATCAGTTACACTCTGTGTATAGATGATCTACTTAGCTATTTGTGTTGATTCTTAGC
TTTCAAAGGTTGCTTTCAAATATTAGCTTTTAGCCTTTCAGGTACTGCACATATACATCTTTTGGCCTTTCAGGTTACTGCACATACATAT
GTTTATGGACTTACCAATAGTGACAAACCTTATTGTATTACTATATTAAAATTTGTGCAGTTTTAGATGGTGCACAAAACAAGAACAACAA
ATCTTAGCGCAAAAACGAAGTCATATTTATATTAAACCCAACGGAGAGGGGAACCCCTGTTGTCAGTTATTCTATGGAGTTTTCTTGGG
GGACCATTTCACTTATTTTAAGTTTGACTTTGACTACTGATACACCCGACCTATATTAATTACATGAAGTTACAAACCTTTTTTTTTTTTTTT
TTGATAAAAAGAAATTACAAATCTTTATCTAATTTTTTTTCAGAATTTTCTTTTCAACTAGGTATGTGAACATACAAAA

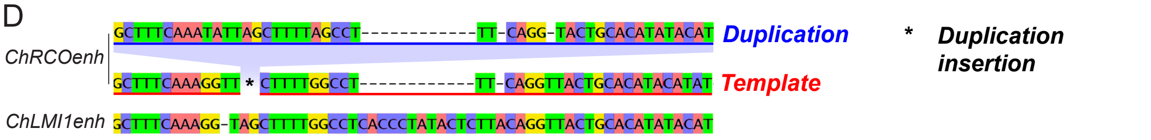

Fig. S9. Sequence analysis of the *RCO* and *LMI1* enhancers. (A) Sequence conservation of 17 *LMI1* and 13 *RCO* enhancer sequences with *ChRCOenh*^{500} computed using a sliding window alignment. The sequences of regions Rep64 and Act69 (the smallest identified deletions conferring either an *RCO* gain-of-function or an *RCO* loss-of-function described in Fig. 1) are highlighted in yellow and green, respectively. **(B)** Detail of the Rep64 region from the alignment reported in (A)

(positions 797 to 941 in the global alignment). Note the conservation of the *RCO* duplication in the *RCO* sequences at the 5’ end of the alignment. **(C)** The *C. hirsuta RCO* enhancer sequence. The sequences of regions Rep64 and Act69 are highlighted in yellow and green, respectively. Blue letters: duplication within the enhancer. Red letters: likely duplication template (original sequence conserved with *LMI1*). **(D)** Alignment of the template and the duplication in the *C. hirsuta RCO* enhancer (red and blue, respectively, as in C) with the ortholog sequence in the *C. hirsuta LMI1* enhancer.

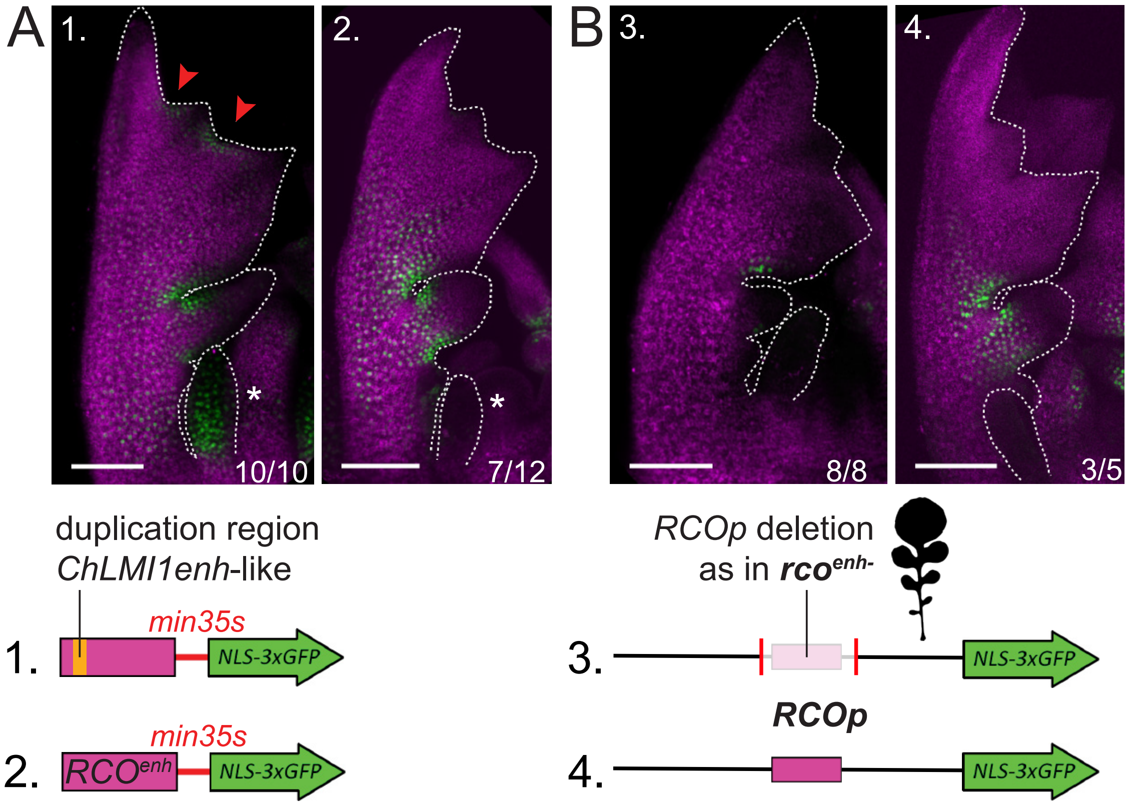

Fig. S10. Expression of *RCOenh*$^{*LMI1\text{-}like\text{-}dupl}$*::NLS:3xGFP* and *pChrco*$^{enh\text{-}}$*::NLS:3xGFP* in *C. hirsuta* leaf primordia. (A) Expression conferred by the *RCO*^{enh} sequence variant (*RCOenh*$^{*LMI1\text{-}like\text{-}dupl}$) where the 'within enhancer' duplication region (S9C-D) is swapped with its *LMI1* enhancer counterpart (S9D) which is a singleton, and drives expression of a *min35s:NLS-3xGFP* cassette. Expression of *RCO*^{enh} WT is also shown for comparison. Red arrowheads show the ectopic expression in the terminal leaflet sinus. Asterisks indicate stipules (note the expression in stipules driven by *RCOenh*$^{*LMI1\text{-}like\text{-}dupl}$*::NLS:3xGFP*). **(B)** Deleting the *rco* enhancer region in an *pRCO::NLS:3xGFP* transcriptional reporter gene such that it recapitulates the *rco*$^{enh\text{-}}$ allele mutation (Fig. 1C) almost completely abolishes gene expression in *C. hirsuta* leaf primordia. Expression of WT *RCO* promoter is also shown for comparison. (A) and (B): the number of independent transgenic lines showing the reported expression profile is indicated at the bottom right of each panel. Green: GFP signal. Magenta: Chlorophyll autofluorescence. Scale bar: 100 mm.

#pattern name	Family	sequence name	Start	Stop	Strand	Score	p-value	q-value	matched sequence
AT4G24020	Nin-like	Rep64	20	34	+	165.625	8.89e-08	8.89e-06	TTTTGGCCTTTCAGG
AT4G01500	B3	Rep64	25	40	+	112.344	7.05e-05	0.00691	GCCTTTCAGGTTACTG
AT5G60970	TCP	Act69	18	25	+	155.769	7.32e-06	0.000823	GGGACCAT
AT3G02150	TCP	Act69	15	27	+	110.548	1.15e-05	0.00116	TGGGGGACCATTT
AT5G08070	TCP	Act69	15	25	-	119.041	1.17e-05	0.00122	ATGGTCCCCCA
AT1G53230	TCP	Act69	15	27	+	118.906	1.21e-05	0.00125	TGGGGGACCATTT
AT1G29860	WRKY	Act69	40	50	-	134.062	1.79e-05	0.00193	CAAAGTCAAAC
AT4G18390	TCP	Act69	16	25	+	127.447	1.92e-05	0.000886	GGGGGACCAT
AT4G18390	TCP	Act69	16	25	-	127.447	1.92e-05	0.000886	ATGGTCCCCC
AT5G26170	WRKY	Act69	40	52	+	131.875	2.01e-05	0.00211	GTTTGACTTTGAC
AT1G30210	TCP	Act69	15	26	+	114.844	2.01e-05	0.00215	TGGGGGACCATT
AT2G21900	WRKY	Act69	46	57	-	131.781	2.06e-05	0.00216	CAGTAGTCAAAG
AT3G01970	WRKY	Act69	40	51	+	126.094	2.24e-05	0.00249	GTTTGACTTTGA
AT5G46350	WRKY	Act69	38	52	-	115.469	2.52e-05	0.00265	GTCAAAGTCAAACTT
AT4G18170	WRKY	Act69	38	50	+	12.875	2.67e-05	0.00299	AAGTTTGACTTTG
AT2G30590	WRKY	Act69	40	50	-	123.594	2.73e-05	0.00296	CAAAGTCAAAC
AT1G80840	WRKY	Act69	38	52	+	128.594	2.75e-05	0.00296	AAGTTTGACTTTGAC
AT2G03340	WRKY	Act69	40	50	-	128.594	2.8e-05	0.0031	CAAAGTCAAAC
AT2G38470	WRKY	Act69	40	50	-	128.906	2.89e-05	0.0031	CAAAGTCAAAC
AT2G23320	WRKY	Act69	40	50	-	12.625	3.2e-05	0.00343	CAAAGTCAAAC
AT4G01250	WRKY	Act69	38	50	-	11.875	3.43e-05	0.0037	CAAAGTCAAACTT
AT5G52830	WRKY	Act69	38	50	+	107.031	3.52e-05	0.00382	AAGTTTGACTTTG
AT4G24240	WRKY	Act69	38	51	+	98.125	3.59e-05	0.00374	AAGTTTGACTTTGA
AT4G23550	WRKY	Act69	40	50	-	124.062	3.64e-05	0.00414	CAAAGTCAAAC
AT4G31550	WRKY	Act69	38	51	+	114.531	3.86e-05	0.00337	AAGTTTGACTTTGA
AT2G46130	WRKY	Act69	44	57	+	115.469	4.47e-05	0.00461	GACTTTGACTACTG
AT4G26640	WRKY	Act69	44	56	+	12	4.75e-05	0.00456	GACTTTGACTACT
AT2G46400	WRKY	Act69	46	56	-	111.918	5.14e-05	0.00413	AGTAGTCAAAG
AT3G15030	TCP	Act69	18	25	+	860.638	5.14e-05	0.0057	GGGACCAT
AT5G13080	WRKY	Act69	44	56	-	113.906	5.15e-05	0.00321	AGTAGTCAAAGTC
AT2G21900	WRKY	Act69	40	51	-	120.548	5.72e-05	0.003	TCAAAGTCAAAC
AT1G35560	TCP	Act69	15	24	-	838.793	5.88e-05	0.00636	TGGTCCCCCA
AT1G62300	WRKY	Act69	46	64	+	109.118	6,00E-05	0.00549	CTTTGACTACTGATAC ACC
AT5G13080	WRKY	Act69	38	50	-	110.312	6.1e-05	0.00321	CAAAGTCAAACTT
AT4G18170	WRKY	Act69	44	56	+	116.406	6.18e-05	0.00345	GACTTTGACTACT
AT4G31550	WRKY	Act69	44	57	+	103.594	6.19e-05	0.00337	GACTTTGACTACTG

AT5G07100	WRKY	Act69	38	50	-	105.063	6.34e-05	0.00338	CAAAGTCAAACTT
AT5G07100	WRKY	Act69	44	56	-	105.063	6.34e-05	0.00338	AGTAGTCAAAGTC
AT2G30250	WRKY	Act69	40	50	-	115.156	6.53e-05	0.00396	CAAAGTCAAAC
AT2G38470	WRKY	Act69	46	56	-	115.469	6.56e-05	0.00351	AGTAGTCAAAG
AT2G24570	WRKY	Act69	38	51	-	101.096	6.59e-05	0.00393	TCAAAGTCAAACTT
AT2G23320	WRKY	Act69	46	56	-	11.375	6.65e-05	0.00356	AGTAGTCAAAG
AT2G47260	WRKY	Act69	46	53	-	120.319	6.72e-05	0.00765	AGTCAAAG
AT2G03340	WRKY	Act69	46	56	-	112.812	7,00E-05	0.00387	AGTAGTCAAAG
AT1G30650	WRKY	Act69	38	50	-	100.469	7.01e-05	0.00484	CAAAGTCAAACTT
AT5G04390	C2H2	Act69	22	32	-	108.594	7.04e-05	0.00791	AAGTGAAATGG
AT4G23550	WRKY	Act69	46	56	-	11.125	7.39e-05	0.00421	AGTAGTCAAAG
AT2G30250	WRKY	Act69	46	56	-	111.875	7.41e-05	0.00396	AGTAGTCAAAG
AT2G46400	WRKY	Act69	40	50	-	104.932	7.46e-05	0.00413	CAAAGTCAAAC
AT2G24570	WRKY	Act69	44	57	-	967.123	7.85e-05	0.00393	CAGTAGTCAAAGTC
AT4G22070	WRKY	Act69	46	64	-	985.294	7.86e-05	0.00727	GGTGTATCAGTAGTC AAAG
AT1G29280	WRKY	Act69	46	56	-	10.125	8.36e-05	0.00898	AGTAGTCAAAG
AT3G13040	G2-like	Act69	7	15	-	117.671	8.53e-05	0.00981	AAGAAAACT
AT4G26640	WRKY	Act69	38	50	+	107.812	8.59e-05	0.00456	AAGTTTGACTTTG
AT3G01970	WRKY	Act69	46	57	+	929.688	8.75e-05	0.00486	CTTTGACTACTG
AT5G52830	WRKY	Act69	44	56	+	801.562	8.82e-05	0.00478	GACTTTGACTACT
AT2G30590	WRKY	Act69	46	56	-	959.375	8.91e-05	0.00482	AGTAGTCAAAG
AT1G30650	WRKY	Act69	44	56	-	93.125	9.05e-05	0.00484	AGTAGTCAAAGTC
AT4G01250	WRKY	Act69	44	56	-	09.05	9.17e-05	0.00495	AGTAGTCAAAGTC
AT1G67260	TCP	Act69	15	44	+	21.519	9.25e-05	0.0074	TGGGGGACCATTTCA CTTATTTTAAGTTTG
AT1G29860	WRKY	Act69	46	56	-	100.781	9.29e-05	0.00502	AGTAGTCAAAG
AT1G47870	E2F/DP	Act69	13	27	-	576.596	9.37e-05	0.00981	AAATGGTCCCCCAAG
AT5G46350	WRKY	Act69	44	58	-	748.438	9.54e-05	0.00502	TCAGTAGTCAAAGTC
AT5G51910	TCP	Act69	16	25	+	103.298	9.77e-05	0.0103	GGGGGACCAT
AT2G46130	WRKY	Act69	38	51	+	985.938	9.81e-05	0.00506	AAGTTTGACTTTGA

Table S11. Predicted transcription factor binding sites in the Rep64 and Act69 regions. Predictions were obtained using the PlantRegMap Binding Site Prediction tool, with the *A. thaliana* Transcription Factor (TF) motif database. A p-value cutoff of 1e-4 was used. Binding sites identified within Rep64 and Act69 are highlighted in yellow and green, respectively.

Supplementary text 1: Discussion on cases of mismatch of allelic effects on phenotype and gene expression as measured by qRT-PCR or reporter gene assays.

Using a combination of qRT PCR and reporter gene analysis of genome edited alleles we showed that the *RCO* enhancer is subject to more negative regulation than its ancestral *LMI1* counterpart, and that *LMI1* enhancer mutations readily produce alleles that yield reduced transcript levels and loss-of-function phenotypes (see main text and Figs. 1-2). However not all alleles we recovered showed a full correspondence between phenotype, qRT-PCR, and reporter gene data (Fig. 4). For ***mut4***, a weak *rco* loss-of-function phenotype (Fig. 1D) coincides with reduced GFP reporter signal (Fig. 4E') but not with a measurable decrease in qRT-PCR (Fig. 1E) suggesting that qRT-PCR may miss subtle changes restricted to specific cells or developmental stages, particularly as with imaging we analyzed a single leaf at a defined developmental stage, whereas qRT-PCR on seedlings evaluates expression in a broader pool of tissue. ***mut3*** shows increased *RCO* transcript abundance (Fig. 1E) and a trend toward greater leaf dissection, but the effect was not statistically significant (Fig. 1D), perhaps because increased or broadened expression does not occur in developmental domains or stages where it is sufficient to drive a strong phenotype. ***mut15*** shows weak *rco* loss-of-function phenotype (reduced petiolule formation; Fig. 3D) without detectable changes in *RCO* transcript levels by qRT-PCR (Fig. 3C). A reason for this might, again, be localized or cell-type-specific gene expression changes that drive the phenotype.

Reporter gene imaging of ***mut14*** indicates higher *RCO* expression than in ***mut5,*** which like ***mut14*** shows broadened expression relative to the wild type (Fig. 4F′ vs. Fig. 4D′), yet qRT-PCR shows both alleles are similarly upregulated (~10-fold; Figs. 1E, 3C). This discrepancy may reflect the reporter gene capturing only a subset of the native expression pattern perhaps because additional sequences not included in the reporter gene construct are important, as well as position effects and copy number differences of transgene insertion.

Resolving such genotype–phenotype mismatches will require expanding the set of transgenic reporter lines and performing additional ISH assays in more alleles. Yet both approaches have inherent limitations—transgenic assays are sensitive to insertion site, and ISH is typically not quantitative and has less resolution than reporter gene assays. Consequently, while our approach yields valuable information in terms of linking genotype to phenotype, a full correspondence of transcript level and distribution, reporter gene expression and phenotype may not be possible to attain for all alleles. Developing improved knock-in strategies that readily allow allele-specific gene expression analysis in the native genomic context using fluorescent tags will be an important next step in resolving these issues.

Dataset S1. Alignment of the *RCO* enhancer sequences of the mutants reported in Fig. 1 and the WT (between -1555bp and -478bp from *RCO* ATG).

Dataset S2. Alignment of the *LMI1* enhancer sequences of the mutants reported in Fig. 2 and the WT (between -2709bp and -631bp from *LMI1* ATG).

Dataset S3. Alignment of the *lmi1*$^{-}$ mutant sequence and the WT (between -3703bp before *LMI1* ATG and 90bp after *LMI1* stop codon).

Dataset S4. Alignment of the sequences of *mut14*, *mut15* and the WT (from 2708bp upstream *LMI1* ATG to 478bp upstream *RCO* ATG).

Dataset S5. Alignment performed on 14 *RCO* and 17 *LMI1* enhancer sequences (see Bioinformatics, quantification and statistical analysis).

SI References

1. A. Hay, M. Tsiantis, The genetic basis for differences in leaf form between *Arabidopsis thaliana* and its wild relative *Cardamine hirsuta*. *Nature genetics* **38**, 942-947 (2006).
2. W. Yan, D. Chen, K. Kaufmann, Efficient multiplex mutagenesis by RNA-guided Cas9 and its use in the characterization of regulatory elements in the AGAMOUS gene. *Plant Methods* **12**, 23 (2016).
3. F. Fauser, S. Schiml, H. Puchta, Both CRISPR/Cas-based nucleases and nickases can be used efficiently for genome engineering in Arabidopsis thaliana. *The Plant journal : for cell and molecular biology* **79**, 348-359 (2014).
4. M. Labuhn *et al.*, Refined sgRNA efficacy prediction improves large- and small-scale CRISPR-Cas9 applications. *Nucleic Acids Res* **46**, 1375-1385 (2018).
5. M. Stemmer, T. Thumberger, M. Del Sol Keyer, J. Wittbrodt, J. L. Mateo, CCTop: An Intuitive, Flexible and Reliable CRISPR/Cas9 Target Prediction Tool. *PLoS One* **10**, e0124633 (2015).
6. M. Hajheidari *et al.*, Autoregulation of RCO by Low-Affinity Binding Modulates Cytokinin Action and Shapes Leaf Diversity. *Current biology : CB* 10.1016/j.cub.2019.10.040 (2019).
7. Y. Wang, N. Bhatia, M. Tsiantis, A suppressor screen of an Arabidopsis thaliana REDUCED COMPLEXITY (RCO)-expressing strain provides insight into the genetics of leaf margin complexity. *The Plant journal : for cell and molecular biology* **122**, e70278 (2025).
8. Z. Zhang *et al.*, A WOX/Auxin Biosynthesis Module Controls Growth to Shape Leaf Form. *Current biology : CB* **30**, 4857-4868.e4856 (2020).
9. P. N. Benfey, L. Ren, N. H. Chua, Tissue-specific expression from CaMV 35S enhancer subdomains in early stages of plant development. *EMBO J* **9**, 1677-1684 (1990).
10. S. J. Clough, A. F. Bent, Floral dip: a simplified method for Agrobacterium-mediated transformation of *Arabidopsis thaliana*. *The Plant journal : for cell and molecular biology* **16**, 735-743 (1998).
11. K. J. Livak, T. D. Schmittgen, Analysis of relative gene expression data using real-time quantitative PCR and the 2(-Delta Delta C(T)) Method. *Methods* **25**, 402-408 (2001).
12. D. Vlad *et al.*, Leaf Shape Evolution Through Duplication, Regulatory Diversification, and Loss of a Homeobox Gene. *Science (New York, N.Y.)* **343**, 780-783 (2014).
13. J. Schindelin *et al.*, Fiji: an open-source platform for biological-image analysis. *Nat Methods* **9**, 676-682 (2012).
14. A. Leigh, S. Sevanto, J. D. Close, A. B. Nicotra, The influence of leaf size and shape on leaf thermal dynamics: does theory hold up under natural conditions? *Plant Cell Environ* **40**, 237-248 (2017).
15. B. D. Redelings, BAli-Phy version 3: model-based co-estimation of alignment and phylogeny. *Bioinformatics* **37**, 3032-3034 (2021).
16. C. Mayor *et al.*, VISTA : visualizing global DNA sequence alignments of arbitrary length. *Bioinformatics* **16**, 1046-1047 (2000).

17. R. Balkunde *et al.*, Identification of the Trichome Patterning Core Network Using Data from Weak ttg1 Alleles to Constrain the Model Space. *Cell Rep* **33**, 108497 (2020).
18. A. P. Gleave, A versatile binary vector system with a T-DNA organisational structure conducive to efficient integration of cloned DNA into the plant genome. *Plant molecular biology* **20**, 1203-1207 (1992).
19. H. B. Singmann, B.; Westfall, J.; Aust, F.; Ben-Shachar, M.S.; Højsgaard, S.; Fox, J.; Lawrence, M.A.; Mertens, U.; Love, J.; Lenth, R.; Bojesen Christensen, R.H. (2025) afex: Analysis of Factorial Experiments.
20. H. A. Pagès, P.; Gentleman, R.; DebRoy, S.; Carey, V.; Delhomme, N.; Ernst, F.; Huber, W.; Kanali, B.; Khan, H.; Lakshman, A.; O'Neill, K.; Obenchain, V.; Ramos, M.; Vill, A.; Wokaty, J.; Wright, E. (2025) Efficient manipulation of biological strings.
21. H. Wickham (2009) ggplot2: Elegant Graphics for Data Analysis. in *Ggplot2: Elegant Graphics for Data Analysis*, pp 1-212.
22. F. Tian, D. C. Yang, Y. Q. Meng, J. Jin, G. Gao, PlantRegMap: charting functional regulatory maps in plants. *Nucleic Acids Res* **48**, D1104-d1113 (2020).
23. X. Gan *et al.*, The Cardamine hirsuta genome offers insight into the evolution of morphological diversity. *Nature plants* **2**, 16167 (2016).
